# Watching the grass grow: does recreational cannabis legalization affect retail and agricultural wages?

**DOI:** 10.1186/s42238-022-00149-6

**Published:** 2022-07-26

**Authors:** Sichao Jiang, Keaton Miller

**Affiliations:** Department of Economics, University of Oregon, Eugene, 97403 OR USA

**Keywords:** Cannabis legalization, Policy change, Labor market, Wages, D00, I18, I28, J21, Q10

## Abstract

**Background:**

Over the past several years, cannabis has become legal for recreational use in many US states and jurisdictions around the world. The opening of these markets has led to the establishment of hundreds of cannabis production and retail firms with accompanying demand for labor, leading to concerns about spillover effects on wages from incumbents.

**Methods:**

We study the markets for agricultural and retail labor in Washington and Colorado from 2000 to 2019 using differences-in-differences with synthetic controls. We employ employment data from the Quarterly Census of Employment and Wages, state-level demographic data from the US Census Bureau, and agricultural data from the National Agricultural Statistics Service. We use the least absolute shrinkage and selection operator (LASSO) for variable selection and classification and regression trees (CART) for chained imputation of missing values.

**Results:**

We find little-to-no evidence of a significant difference in weekly wages per worker generated by cannabis legalization: the log of the weekly wage per worker decreases by 0.013 in Washington’s agricultural sector (*p* value 0.091) and increases by 0.059 in Washington’s retail sector (*p* value 0.606). Results in Colorado are qualitatively similar. These results are limited in part by the short post-legalization period of the data.

**Conclusions:**

Cannabis legalization is unlikely to negatively impact incumbent agriculture or retail firms through the labor market channel.

## Introduction

The long-standing landscape of cannabis prohibition is rapidly changing. In the past decade, the median American voter moved from opposing to supporting legalization (Motel 2015), more than a dozen US states legalized the substance for adult use, and jurisdictions around the world loosened restrictions. One argument employed by supporters of legalization is the assertion that policy liberalization would lead to the creation of new jobs across multiple sectors (see, e.g., Keys (2020), Wallace (2020)). Indeed, according to Statistics Canada, the industry generated over 10,000 jobs within a year of Canada’s federal-level legalization, with average hourly wages above the national average, and Barcott and Whitney (2019) estimate that the US cannabis industry (including both medical and adult-use cannabis) directly employed more than 200,000 workers in 2019.

Cannabis, however, does not exist in a vacuum—the labor involved in cannabis production and retail is similar to that involved in other agricultural and retail markets and so cannabis legalization may induce workers to substitute between employers. Indeed, farmers of other crops in many areas have expressed concerns about the potential for upward pressure on agricultural labor wages as a consequence of adult-use cannabis laws (RCLs) (Stoicheff 2018; Smith et al. 2019; Valachovic et al. 2019; Washburn 2020). In this paper, we investigate these concerns by measuring the impact of recreational cannabis legalization on wages using data collected from the US Census Bureau. We focus on Washington and Colorado due to their early adoption of legalization policies and therefore the longest post-legalization period during which to measure any changes in labor markets. We focus on agricultural and retail labor markets as those are plausibly the most likely to be affected by the opening of adult-use cannabis markets.

While this policy change may seem like a relatively clean quasi-experiment—both Washington and Colorado legalized adult-use through ballot initiatives and while the opportunity to generate tax revenue likely played a role in the success of these efforts, it is unlikely that the timing of these ballot initiatives or their implementation was driven by labor market conditions—and an opportunity for a differences-in-differences approach, we must overcome a number of challenges.

The first is data-related: cannabis is not separately categorized by the North American Industry Classification System (NAICS) and so we cannot measure the level of employment in the cannabis industry directly, but must instead infer it from changes in some larger category. Using data from the Quarterly Census of Employment, we identify NAICS categories that experience changes in the number of firms and employees that match state regulator data on cannabis firms. These categories differ across states as a consequence of differing regulatory frameworks. These data limitations create potential limitations in our ability to answer questions: if we observe a large increase in wages in the NAICS categories which contain cannabis firms, we cannot be certain that those higher wages are being paid to other workers in those categories without either additional assumptions or additional data. We address this in part by defining broader categories of retail and agriculture firms over which cannabis firms play a small role; if we observe an increase in wages in these broader categories, we can more reasonably conclude that incumbent firms are paying higher wage bills.

Second, given the spillover effects of legalization efforts both in terms of geography (Hansen et al. 2020a) and in product space (Miller and Seo 2021), as well as the mobility of (particularly agricultural) labor (Thomas-Lycklama-Nijeholt 2012; Holmes 2013), it is difficult to choose an appropriate control group a priori. We therefore follow Hansen et al. (2020b), who study the impact of cannabis legalization on traffic fatalities, and use a synthetic control approach. We create a control group by choosing weights for states without legal cannabis markets to match moments characterizing each state in the pre-legalization period. By comparing post-legalization employment and wages in the treated states to their synthetic controls, we can estimate the causal impact of legalization on these outcomes of interest.

Implementing this approach for the retail sector is relatively straightforward—the elements of retail sectors which drive labor market outcomes (i.e., household income and population density) do so in a consistent way across states (Neumark et al. 2008; Blakely and Leigh 2013). Agricultural sectors in different states, however, are significantly different due to variation in growing conditions and the characteristics of arable land. While many detailed industry measures are available, the set of measures changes frequently and often are not available for all states. Faced with a need to both select variables and impute certain values, we follow the approach of White et al. (2018) and implement machine learning techniques to accomplish these tasks algorithmically. In particular, we use LASSO for variable selection and classification and regression trees (CART) to impute missing values.

Our primary finding is a null result: we find little evidence of a significant difference in weekly wages per worker in the most directly substitutable NAICS categories. Furthermore, though our estimates are noisier, we do not find evidence of changes in weekly wages per worker in our broader definitions of the retail and agricultural sectors.

This paper adds to the growing literature investigating the legalization of cannabis for adult (recreational) use and its effects on outcomes thought to be related to cannabis consumption. Smart and Pacula (2019) summarizes many of the policy implications of cannabis legalization. Specific examples include studies on student performance (Miller et al. 2017), traffic fatalities (Aydelotte et al. 2017; Hansen et al. 2020b), crime (Dragone et al. 2019; Hughes et al. 2020; Hao and Cowan 2020) and the consumption of other “sin” goods and cannabis substitutes (Kerr et al. 2017; Baggio et al. 2018; Miller and Seo 2021; Hansen et al. 2020; Chan et al. 2020).

Our analysis hinges on the assumption that labor supply conditions are largely unaffected by cannabis legalization. Since all states which have legalized cannabis for adult use have previously legalized cannabis for medical use, the effects of both policies are relevant to our study. Ullman (2017) finds that medical cannabis laws (MCLs) reduce the number of absences due to sickness, while Sabia and Nguyen (2018) employ a synthetic control approach and find “no evidence that [MCLs] affect employment, hours, or wages among working-age adults,” Nicholas and Maclean (2019) find evidence that MCLs “lead to increases in older adult labor supply, with effects concentrated on the intensive margin” and Ghimire and Maclean (2020) provide evidence that workers’ compensation claims fall following the adoption of MCLs. On the adult-use side, Maclean et al. (2021) argue that RCLs increase Social Security disability claims, while Abouk et al. (2021) find that workers’ compensation benefits decline after RCL adoption. Taken together, these results suggest that our assumption is reasonable to a first-order approximation, though we discuss the way in which increases in labor supply driven by RCL adoption would influence our results in our conclusion.

More recently, the literature has begun to examine the cannabis industry as an economic entity of interest in and of itself and as a tool to investigate long-standing questions in industrial organization and policy design: Hansen et al. (2017) investigate the impact of a change in Washington’s tax structure throughout the cannabis supply chain, Thomas (2018) considers the effect of Washington’s licensing quota system, Hollenbeck and Uetake (2021) estimate the level and effects of market power in the industry, and Berger and Seegert (2020) use the cannabis industry to analyze the effects of financial exclusion on firms.

Within the literature, the closest effort to that of our own is that of Chakraborty et al. (2020), who study the effects of Colorado’s legalization on labor market outcomes at the county level exploiting the timing of retail entry across counties. Ultimately, they find, as we do, that while the entry of legal cannabis employers leads to increases in the number of employees in the relevant sectors, the impact on equilibrium wages is approximately zero. Relative to that work, we aggregate to the state level to avoid concerns about intra-state labor mobility, use states without legal cannabis markets as the bases for synthetic controls to avoid inter-state spillover effects, and add an additional treated unit (Washington).

We proceed in the “Labor in the cannabis industry” section by describing labor in the cannabis industry relative to other agricultural and retail industries. In the “Data and methodology” section, we describe our data on labor market outcomes and our methodology. In the “Results” section, we present our findings. We conclude in the “Conclusion” section with a discussion of the policy implications and suggestions for future research.

## Labor in the cannabis industry

Relative to many commodity agriculture crops such as corn and wheat, cannabis production is labor intensive owing in large part of the dioecious nature of plants in genus *Cannabis*. Buds with high concentrations of the psychoactive cannabinoids tetrahydrocannabinol (THC) and cannabidiol (CBD) (among others) are only produced by female plants prior to pollination (Chandra et al. 2017). Thus, in contrast to other dioecious agriculture operations, such as fruiting trees where males are necessary for fruit production, cannabis growers must identify and remove male cannabis plants from growing areas as even a small number of male plants can provide pollen for an entire crop, triggering seed production in females, a diminished set of flowers, and a corresponding reduction in cannabinoid production. This labor is necessary even when farmers plant “feminized” seeds or clones of female plants as the costs of a single male plant are high enough that growers use labor resources to identify and destroy male buds (see, e.g., Schaneman 2019). A relevant analogy in traditionally-legal agricultural products is hops (*Humulus lupulus*); producers of hops remove male plants to prevent pollination (Shepard et al. 1999).

The prevalence of indoor growing facilities complicates direct comparisons between cannabis and other plants. According to an industry report, 60% of legal producers operate indoor facilities, and 41% operate greenhouses—only 12% of firms grow cannabis in the outdoors alone (Cannabis Business Times 2020). The use of indoor and greenhouse spaces allows for more precise control of the growing environment, leading to more potent output (Aizpurua-Olaizola et al. 2016), and enables production regardless of the outdoor agricultural season. However, the amount of labor hours needed per pound produced is likely higher for indoor and greenhouse operations than for outdoor operations (Caulkins 2010).

After budding, plants must be harvested and trimmed of buds—a process which takes four to six hours per pound manually (Cervantes 2006). While mechanized trimmers are available, hand-trimmers are able to extract higher quality buds from plants which can command higher prices from consumers; the majority of products sold to consumers (by revenue) consists of dried and cured buds and thus the visual appearance of the buds is directly relevant to demand (Miller and Seo 2021). The remaining plant material undergoes extraction processes to produce concentrate and edible products which are generally sold at a lower price per weight of plant input. As a consequence, skilled trimmers can earn more than twice the average hourly wage of other laborers in crop, nursery, and greenhouse operations (Krissman 2017).

These features of the cannabis industry imply that it is at least plausible that a small number of cannabis producers (relative to the number of other agricultural producers using greenhouses) could sufficiently impact the aggregate demand for agricultural labor to significantly change equilibrium wages. However, relative to other agricultural products, the market for cannabis labor is tightly regulated. In each state with an operating recreational market, individuals must pass a background check before working for a cannabis producer—and to pass that check, the worker must have legal immigration status and (in most states) must not have recent felony convictions related to Schedule I or Schedule II drugs. According to the US Department of Labor, approximately 47% of the US agricultural labor industry are undocumented immigrants, though agricultural industry sources estimate the share is closer to 75% (Jordan 2020). If the labor markets are bifurcated due to immigration status, the effects of legalization on wages may be minimal at best. Furthermore, as the highest wages available within the cannabis industry are paid to workers with cannabis-specific skills, the substitutability of that labor (and therefore the upwards pressure on equilibrium wages) may be limited.

The process of retail sales of cannabis products also differ from most retail businesses. In most jurisdictions, psychoactive cannabis inventory must be strictly and securely separated from the sales floor, which is often required to be separated from pedestrian access through secure doors so that customer ages can be verified before entry. Inventory must be tracked in real-time for compliance with federal guidelines and state seed-to-sale traceability regulations. Audits are frequent and penalities for non-compliance include civil and criminal liability for firm owners and managers (Hansen et al. 2018). These additional layers of security and related regulations imply that, relative to other retailers with similar footprints, cannabis retailers may demand additional labor hours.

Finally, though Colorado and Washington set up recreational markets in the same time period, the regulatory structures vary in ways relevant to our analyses; see Hansen et al. (2021a) for more details about the regulatory structures in the various states which have legalized cannabis for adult use. First, while Washington required vertical separation between production and retail, Colorado initially required retailers to produce 70% of the products they sell through vertically integrated production facilities, often located close to the retailer (Hansen et al. 2021b). As a consequence, while firms in both Washington and Colorado set up production operations, production facilities in Washington, which were both more geographically dispersed and more specialized, arguably competed more directly with other greenhouse agricultural facilities for labor. Second, Colorado initially limited adult-use licenses to existing medical dispensaries, which may limit the number of new establishments entering at the time Colorado’s market opened. Finally, Colorado allows home cultivation, which Washington bans. While this may affect demand for cannabis on the margin, we note that to-date, the cannabis industry in Colorado has generated more revenue per resident than Washington’s industry.

## Data and methodology

We begin our analysis of the relationship between cannabis legalization and labor market outcomes by obtaining labor market data from the Quarterly Census of Employment and Wages compiled by the US Bureau of Labor Statistics (BLS). BLS categorizes employers according to the North American Industry Classification System (NAICS)—a system of 2–6 digit codes which classifies employers in narrowing groups according to their output or primary business activity. Our outcomes of interest include the number of establishments, the total number of workers, the total real wages, and the average weekly real wage per worker. We collect these outcomes at the NAICS-state-quarter level from 2000 to 2019, aggregate to the annual level, and deflate to 2019 dollars using the Consumer Price Index.

To capture time-varying characteristics of labor markets which may influence outcomes, we collect demographic data from the US Census Bureau and Department of Education including state-level high school and college graduation rates, population density, the aggregate unemployment rate, and per-capita GDP. Agricultural labor markets differ widely from state to state due to differences in the characteristics of arable land and growing seasons and therefore to capture other time-varying characteristics of agricultural markets which may influence relevant labor market outcomes, we additionally collect state-year-level survey data from the National Agricultural Statistics Service from 2000 to 2015 and state-level data from the US Censuses of Agriculture for 2002, 2007, and 2012 (i.e., pre-treatment covariates). A challenge we face in using this data is the prevalence of missing values which stem in part from changes in the survey questions from year to year. To create a panel data set for analysis, we focus on variables for which there are at least 30 state-level observations per year. These variables largely sort into clear topic areas: demographics, land statistics including rental prices, counts of farm establishments, and variables capturing output for corn, wheat, hay, and fruits and vegetables.

Despite this restriction, the data still contain many missing values complicating any analysis effort. Following White et al. (2012; 2018), we use the Van Buuren et al. (2006) modification of the Classification and Regression Trees (CART) algorithm to impute missing values. The algorithm uses a Gibbs sampling procedure to generate a plausible value for each missing value. Key to our application, the algorithm uses “chained” imputation: for each unit of observation (i.e., each state-year observation), the most recent generated imputation for each column is used as a predictor for the next column to minimize bias (Van Buuren and Groothuis-Oudshoorn 2010; Murray and Reiter 2016; Michalowsky et al. 2020). In other words, suppose the vector of independent variables for observation *t* is *X*_*T*_=[*x*_1*t*_,*x*_2*t*_,⋯ ]. Suppose *x*_1*t*_ is known for some *t* but *x*_2*t*_ is missing. The algorithm uses a Gibbs sampler to draw a value from *x*_2*t*_ using the empirical distribution of *x*_2_ conditional on *x*_1*t*_. Now suppose *x*_3*t*_ is also missing for *t*. The algorithm uses both the observed value *x*_1*t*_ and the imputed *x*_2*t*_ to draw a value of the *x*_3_ distribution conditional on both *x*_1_ and *x*_2_. Ultimately, in our primary specification, we impute 11% of the observation-variables for the agricultural analysis and none of the observation-variables for the retail analysis. We have re-estimated our models excluding imputed data and found similar results.

We next turn to the issue of variable selection. The number of potential control units (i.e., states other than Washington and Colorado) is less than the number of potential covariates. Instead of manually choosing covariates based on some prior hypothesis, which may be considered “cherry picking” (Ferman et al. 2020), we use the LASSO algorithm to select appropriate covariates (Tibshirani 1996; Duncan et al. 2019). For each outcome variable, we fit prediction models to the pre-legalization data (i.e., data from 2000 to 2012) using the glmnet method of Friedman et al. (2010) and select the covariates with the highest frequency for each of the outcome variables.

The final covariate matrix *X* for our agricultural analysis includes “Barley for grain (acres)”; “Land in orchards (acres)”; “Snap beans harvested for sale (acres)”; “Cherries (acres)”; “Pears (acres)”; “Commercial fertilizer, lime, & soil conditioners (acres treated)”; “2000 Resident population 65 years & over, percent”; “2000 Savings institutions (FDIC-insured)-total deposits”; “2000 Civilian labor force unemployment rate”; “Federal Government expenditure-grants FY 2000”; “Federal Government insurance FY 2000”; “2000 Resident population: Black alone, percent”; “2000 Resident population: Two or more races, percent”; “2000 Resident population: Hispanic or Latino Origin, percent”; “2000 Resident population: total females, percent”; “Social security: retired workers-benefit recipients (Dec.) 2000”; “Corn grain production”; “Farm operations”; “Hay production”; “Labor hired wage rate ($ per hour)”; “Rent cash cropland expense ($ per acre)”; “Vegetable total production”; and “Wheat production.” For our retail analyses, the covariate matrix includes “College Graduation Rate (percent)”; “High School Graduation Rate (percent)”; “Population Density (people per square mile)”; “Unemployment Rate (percent)”; and “GDP per capita.” We also include the relevant outcome for stores in NAICS 453991 (Tobacco stores).

The agricultural census data is collected every 5 years—the last collection was in 2017. At the time of the last collection, only four states—Alaska, Colorado, Oregon, and Washington—had legalization cannabis for recreational use, and within those states, Colorado and Washington legalized earliest (voting in 2012, markets opening in 2014). To focus on the longest post-legalization period possible, we follow Hansen et al. (2020b) and focus on Colorado and Washington as the treated states. We further note that both Oregon and Alaska experienced significant supply issues in months immediately post market-opening (Sacirbey 2016; Andrews 2017) and thus any impact on agricultural labor is potentially more difficult to observe and/or interpret from the short post-legalization period available.

**Fig. 1:**
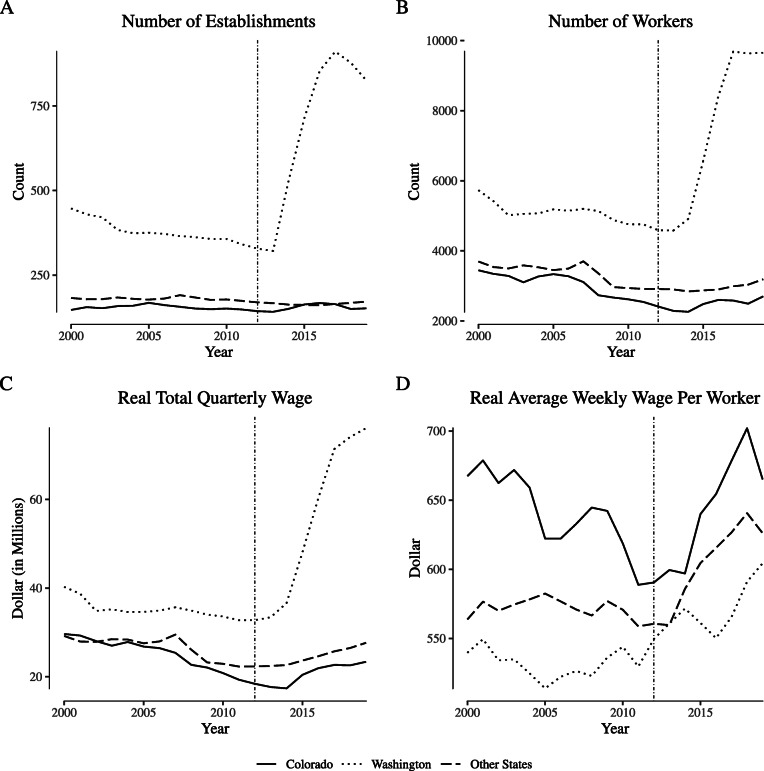
Employment and wages for “narrowly defined” agricultural firms. Notes: Data come from the Quarterly Census of Employment and Wages. We define “narrowly defined” agricultural firms as those within North American Industry Classification System category 1114 (“Greenhouse and Nursery Production”), which includes cannabis production firms

Figure 1 plots outcomes by year for Colorado, Washington, and the average of other states for the “greenhouse, nursery, and floriculture production” category (NAICS 1114, the category containing cannabis production firms). Notably, the establishment count for Washington increased by roughly 500 between legalization and a peak in late 2015, which is similar to the count of cannabis production licenses issued by the state around the same time period as reported by Hansen et al. (2017). Washington experienced a similarly-shaped increase in the number of workers in the sector and the total wages paid, but those outcomes in Colorado and other states remained largely constant. Despite the increase in labor quantity observed in Washington, the real average weekly wage per week increased after legalization relatively uniformly everywhere.

**Fig. 2:**
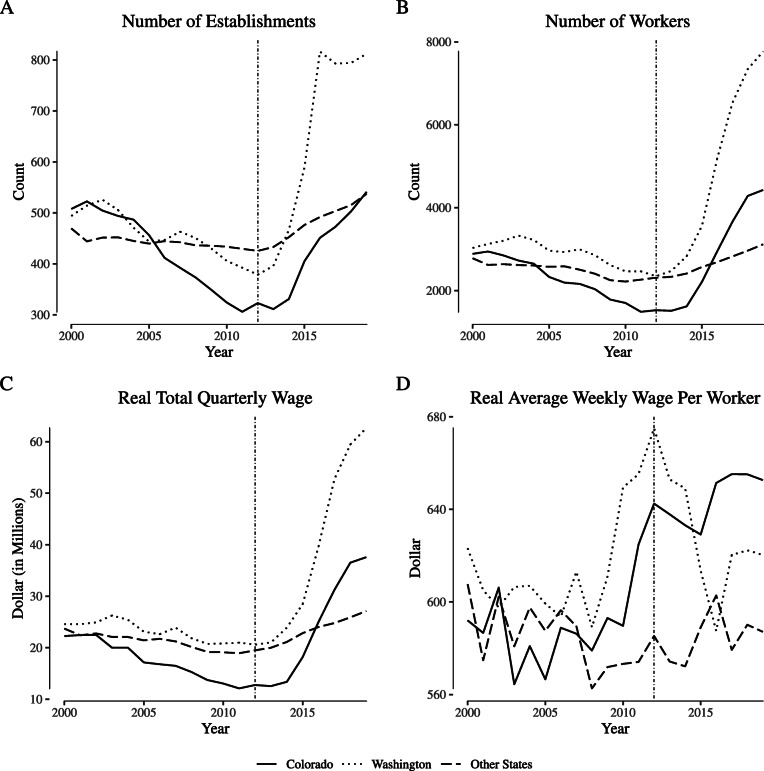
Employment and wages for “narrowly defined” retail firms. Notes: Data come from the Quarterly Census of Employment and Wages. We define “narrowly defined” retail firms as those within North American Industry Classification System category 453998 (“Store retailers not specified elsewhere”), which includes cannabis retailers

Figure 2 reports analogous outcomes in the “store retailers not specified elsewhere” category (NAICS 453998, the category containing cannabis retailers). As with the agricultural sector, the establishment count in Washington increased by several hundred immediately post-legalization corresponding to descriptive statistics found in the literature (Thomas 2018). Colorado also experienced an increase of roughly 200 establishments over the same time period. Increases of similar magnitude occurred for worker counts and total wages paid in conjunction with the opening of these establishments. As in the agricultural sector, however, there are no clear patterns in the average weekly wage per worker; while the mean post-reform wage in Colorado is above the mean pre-reform wage, wages had begun increasing in the years prior to the passage of the ballot measure.

While the raw data suggest that the legalization of cannabis led to significant changes in employment in each state corresponding to their different regulatory structures, it is not clear that cannabis legalization caused these changes. Estimating a causal effect requires identifying an appropriate set of control units. While neighboring states might seem like a natural control group, Hansen et al. (2020a) find evidence of substantial inter-state cannabis demand, and it is reasonable to believe that laborers may also move across state lines in response to cannabis legalization, particularly if cannabis producers are indeed offering higher wages. This is a particular concern for Washington, where many retailers are located close to the Oregon and Idaho borders.

To address this concern, we apply the synthetic control approach of Abadie and Gardeazabal (2003), Abadie et al. (2010, 2015). We construct synthetic control units separately for Washington and Colorado based on pre-legalization data (i.e., the covariates listed above plus the lagged value of the outcome variable) and then estimate the effect of cannabis legalization on our outcomes of interest by calculating the post-legalization difference between the outcomes for our treated states and for our synthetic controls. Our synthetic control units are convex combinations of non-treated states selected in such a way to match the pre-legalization outcomes. In addition to previous work on cannabis legalization and traffic fatalities (Hansen et al. 2020b), the synthetic control approach has been used to analyze the effects of policy changes across a variety of domains, including economic liberalization (Billmeier and Nannicini 2013), pediatric health (Bauhoff 2014), tropical deforestation (Sills et al. 2015), foreign exchange rates (Chamon et al. 2017), tobacco policies (Chelwa et al. 2017), and the effects of medical cannabis laws on labor market outcomes (Sabia and Nguyen 2018) among many others.

We first select a “donor pool” of control units (i.e., states) which may be used to construct the synthetic control units. We start with all US states and exclude any states which legalized cannabis and opened adult-use markets after 2012. We include Michigan as its first dispensary opened in December 2019, and thus any labor market effects are unlikely to be observed in annualized 2019 data. We also exclude states which are adjacent to the treated states to avoid spillover effects. While we present results using a donor pool which includes both states with and without legal medical cannabis markets, we have estimated separate models using only states with or states without these markets and found similar results.

For each treated unit *s*∈{Washington, Colorado }, we then select weights *w*_*j*_ for each of the control units *j* (with 0≤*w*_*j*_≤1 and $\sum w_{j} = 1$) to minimize the weighted difference between the synthetic control and the treated unit on the pre-treatment covariates identified above. The weight matrix *V* used to form the distance measure is chosen such that the mean square prediction error is minimized for the pre-intervention period following Abadie et al. (2010). We report the weights *W*^∗^ chosen for each treated unit and outcome variable in Appendix A. Tables of covariate balance are available in Appendix B. We then obtain point estimates of the effect of recreational cannabis legalization with a standard differences-in-differences estimating equation. For outcome *y* for unit *s* (either a treated state or the synthetic control for that state) in year *t*, we estimate the parameters of

1 $$ \begin{aligned} y_{st} &= \beta_{0} + \beta_{1} * {\text{Legal}}_{t} + \beta_{2} * {\text{Treated}}_{t}\\ &\quad+ \beta_{3} * {\text{Legal}}_{t} * {\text{Treated}}_{t} + \epsilon_{st}. \end{aligned}  $$

To perform hypothesis testing, we use the “in-space” placebo tests described in Abadie et al. (2015). In particular, we apply the synthetic control model to each of our potential control units and interpret the results as placebos. We remove a small number of control states with particularly poor pre-treatment fit, though this does not affect our qualitative results. Plots of these placebos are available in the Appendix. For each outcome *Y* (and corresponding sequence of state-year outcome observations *Y*_*jt*_), we then calculate the empirical distribution of the *ratio of the mean squared prediction errors* (RMSPE) where

2 $$ \text{RMSPE}=\left(\frac{1}{T_{0}} \sum_{t=1}^{T_{0}}\left(Y_{1 t}-\sum_{j=2}^{J+1} w_{j}^{*} Y_{j t}\right)^{2}\right)^{1 / 2}  $$

and *T*_0_ is the positive number of pre-intervention periods. The *p* value is then simply the fraction of placebo effect estimates which are greater than or equal to the effect estimated for the treated unit (Firpo and Possebom):

$$p:=\frac{\sum_{j=1}^{J+1} \mathbbm{1}\left[\operatorname{RMSPE}_{j} \geqslant \operatorname{RMSPE}_{1}\right]}{J+1} $$

Finally, it is plausible that, from the perspective of workers, jobs in the cannabis industry are substitutes for jobs beyond the narrowly-defined NAICS categories described above. We repeat this analysis for a broader set of categories taking advantage of the hierarchical nature of the NAICS inclusive of cannabis firms; for agriculture, we use “agriculture, forestry, fishing, and hunting” (NAICS 11) and for retail, we aggregate the “health and personal care stores” (NAICS 446), “general merchandise stores” (NAICS 452) and “miscellaneous store retailers” (NAICS 453) categories.

## Results

### Narrowly-defined industries

**Fig. 3:**
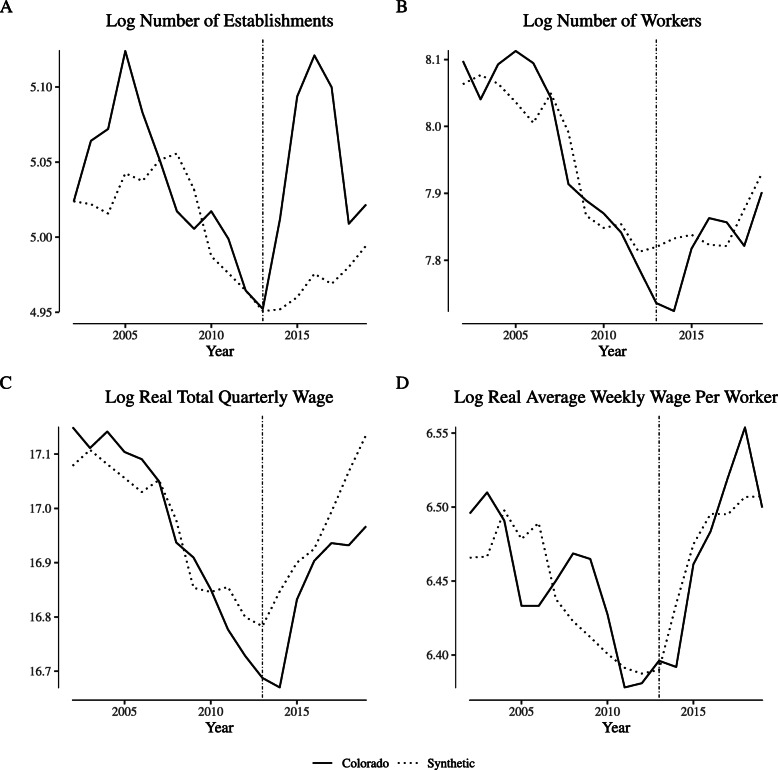
Comparing “narrowly defined” agricultural labor market outcomes in Colorado and its synthetic control. Notes: This figure depicts wage and employment outcomes for “narrowly defined” agricultural firms for Colorado and its synthetic control. We define “narrowly defined” agricultural firms as those within North American Industry Classification System category 1114 (“Greenhouse and Nursery Production”), which includes cannabis production firms

Figure 3 illustrates agricultural labor market outcome measures in Colorado and its synthetic control unit (control weights are reported in Appendix A: Table 4) for the “greenhouse, nursery, and floriculture production” NAICS category. Following Fig. 1, Panel (a) illustrates the log of the number of establishments, Panel (b) illustrates the log of the number of worker, Panel (c) illustrates the log of the real total quarterly wage, and Panel (d) illustrates the log of the real average weekly wage. In general, the synthetic control closely follows both the trends and the level of Colorado’s outcomes over the pre-legalization period. In the post-legalization period, the number of establishments temporarily grows relative to its synthetic control, but the number of workers tracks closely with its synthetic control, as do wages.

**Fig. 4:**
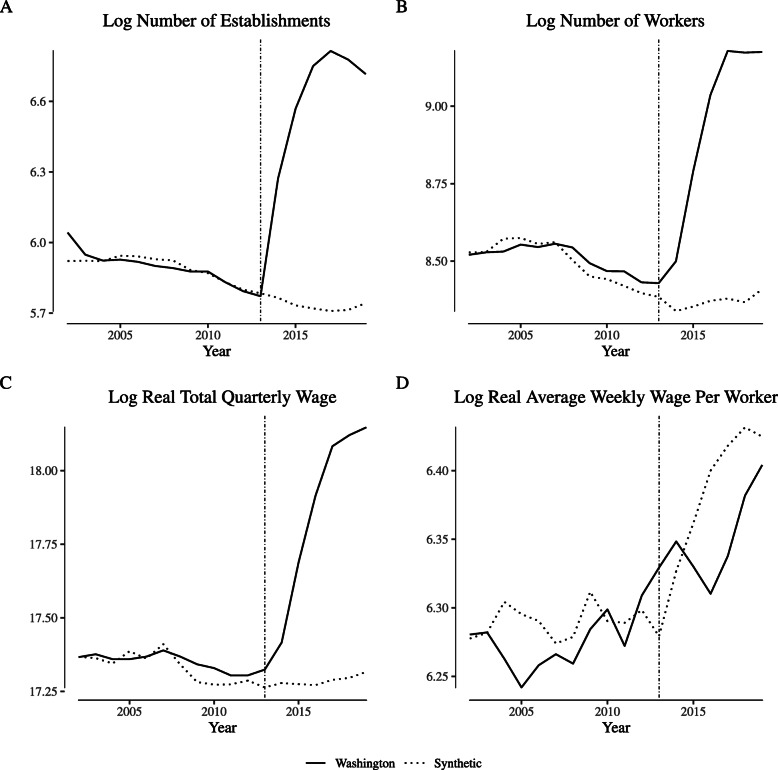
Comparing “narrowly defined” agriculture labor market outcomes in Washington and its synthetic control. Notes: This figure depicts wage and employment outcomes for “narrowly defined” agricultural firms for Washington and its synthetic control. We define “narrowly defined” agricultural firms as those within North American Industry Classification System category 1114 (“Greenhouse and Nursery Production”), which includes cannabis production firms

Figure 4 illustrates the analogous comparisons for Washington. As in Colorado, the synthetic control tracks closely with the Washington data in the pre-legalization period. However, the number of establishments increases significantly immediately after legalization, as does the number of works and (as a consequence), the total quarterly wages paid. Though the average weekly wage in Washington does increase post-legalization, the increase is also seen in the synthetic control.

**Fig. 5:**
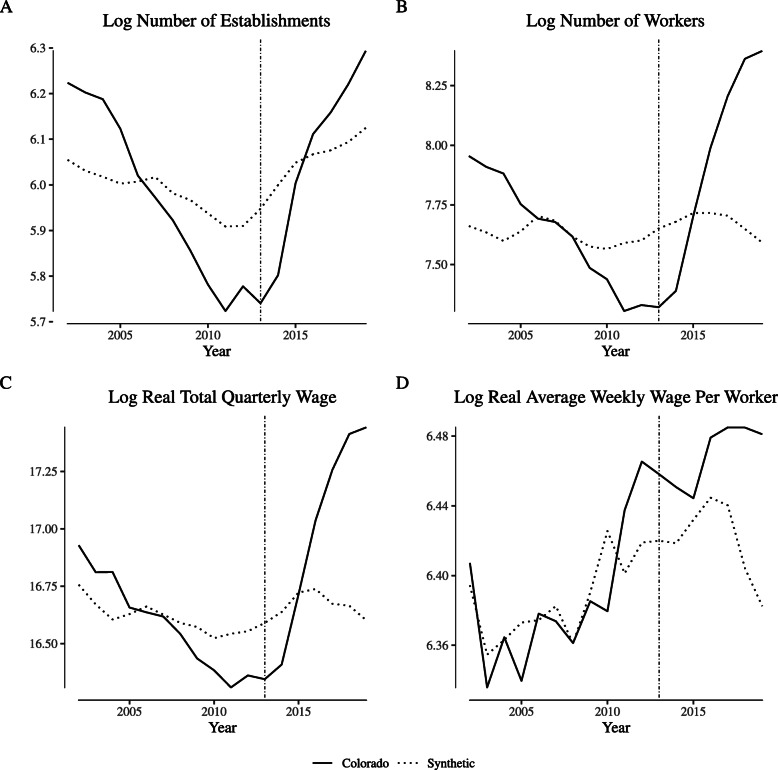
Comparing “narrowly defined” retail labor market outcomes in Colorado and its synthetic control. Notes: This figure depicts wage and employment outcomes for “narrowly defined” retail firms for Colorado and its synthetic control. We define “narrowly defined” retail firms as those within North American Industry Classification System category 453998 (“Store retailers not specified elsewhere”), which includes cannabis retailers

**Fig. 6:**
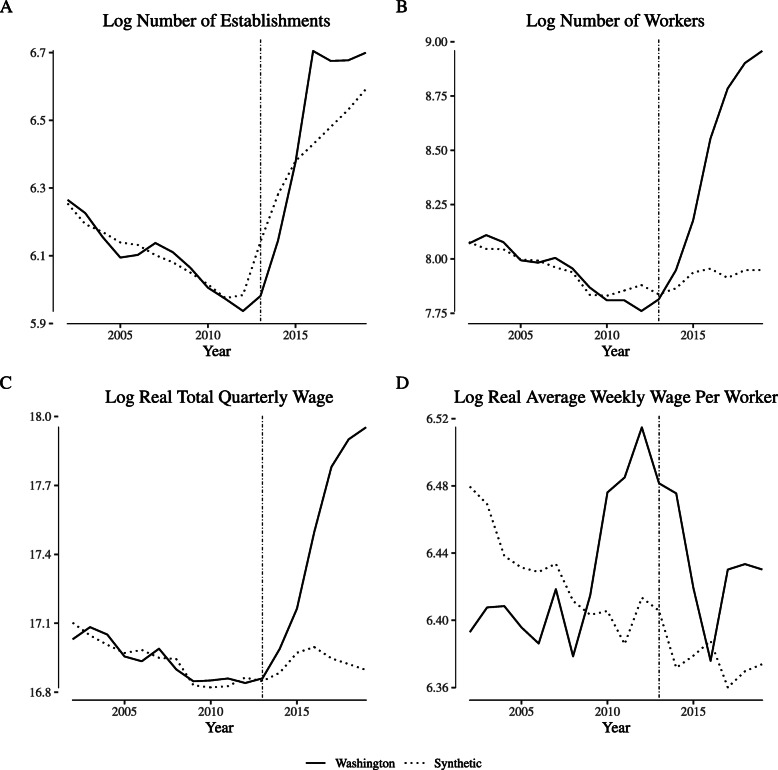
Comparing “narrowly defined” retail labor market outcomes in Washington and its synthetic control. Notes: This figure depicts wage and employment outcomes for “narrowly defined” retail firms for Washington and its synthetic control. We define “narrowly defined” retail firms as those within North American Industry Classification System category 453998 (“Store retailers not specified elsewhere”), which includes cannabis retailers

Figures 5 and 6 repeat the exercise for outcomes for the “store retailers not specified elsewhere” NAICS category in Colorado and Washington, respectively. For Colorado, the synthetic control approach struggles to match the full volatility of the pre-reform data for the number of establishments and the number of workers. However, the method performs better (in a mean-squared-error sense) when matching per-reform average weekly wages per worker. Across outcomes, the synthetic control generally moves in the same direction as the Colorado data post reform, suggesting that other trends in Colorado contributed to the increase in establishments and workers seen in Fig. 2. The synthetic control approach performs better for Washington, where pre-trends are closely matched for most outcomes.

Point estimates of the effects seen in these Figures (i.e., estimates of *β*_3_ in Eq. 1) are reported in Table 1. Several of the changes in the number of establishments, employees, and total wages are significant according to our placebo test at the 10% and 5% levels. However, the change in average weekly wage is either imprecisely estimated or negative for both sectors in both states.

**Table 1:** Synthetic control estimates of the effect of recreational cannabis legalization on narrowly-defined labor market outcomes

	Log number establishments	Log number of employees	Log total quarterly wages	Log weekly wage
**Colorado**				
*Narrowly-defined Agriculture*				
RCL	0.056**	− 0.042	− 0.113	-0.007
*P* value	[0.030]	[0.303]	[0.303]	[0.576]
*Narrowly-defined Retail*				
RCL	0.000	0.220	0.306*	0.050
*P* value	[0.818]	[0.152]	[0.091]	[0.212]
**Washington**				
*Narrowly-defined Agriculture*				
RCL	0.783*	0.516*	0.513**	− 0.013*
*P* value	[0.061]	[0.061]	[0.030]	[0.091]
*Narrowly-defined Retail*				
RCL	0.063	0.535**	0.525**	0.059
*P* value	[0.152]	[0.030]	[0.030]	[0.606]

Notes: This table reports difference-in-difference estimates of the effect of recreational cannabis legalization (RCL) on labor market outcomes using synthetic controls for the treated units. *Agriculture* is the “Greenhouse and Nursery Production” (NAICS 1114) industry. *Retail* is the “Store retailers not specified elsewhere” category (NAICS 453998). *P* values are calculated via a placebo test. Stars indicate standard significance levels: ^∗^10%, ^∗∗^5%, ^∗∗∗^1%

### Broadly-defined industries

While the above results verify that the legalization of cannabis led to changes in the number of establishments and employees working in the categories which contain cannabis firms, they provide no evidence that legalization led to wage spillovers. Indeed, there is little evidence that legalization affected the Colorado labor market at all. One possibility is that although cannabis production facilities are coded as members of the green house and nursery sector, cannabis facilities do not compete with other members of that sector for labor. To explore this possibility, we first repeat the analysis for NAICS 11, which includes all “agriculture, forestry, fishing, and hunting” firms.

The results are reported in Table 2 under the headings for “Broadly-defined Agriculture”—the relevant Figures are available in the Appendix. It is important to note that the pre-treatment fit for Washington is generally poor. Ferman (2021) shows that the synthetic control model can be asymptotically unbiased even when the pre-treatment fit is imperfect. Relative to Table 1, the estimates for Washington are generally attenuated and more noisily estimated. For Colorado, the estimates indicate small and marginally significant increases in employees and total wages, though once again for both states there is no increase in average weekly wages.

**Table 2:** Synthetic control estimates of the effect of recreational cannabis legalization on broadly-defined labor market outcomes

	Log number establishments	Log number of employees	Log total quarterly wages	Log weekly wage
**Colorado**				
*Broadly-defined Agriculture*				
RCL	0.008*	0.108**	0.064**	0.007
*P* value	[0.091]	[0.030]	[0.029]	[0.242]
*Broadly-defined Retail*				
RCL	− 0.044	0.035**	0.055**	0.015*
*P* value	[0.576]	[0.030]	[0.030]	[0.061]
**Washington**				
*Broadly-defined Agriculture*				
RCL	− 0.021	0.312	0.369	-0.154
*P* value	[0.091]	[0.333]	[0.242]	[0.667]
*Broadly-defined Retail*				
RCL	0.013	0.112	0.147*	0.014
*P* value	[0.121]	[0.121]	[0.061]	[0.333]

Notes: This table reports difference-in-difference estimates of the effect of recreational cannabis legalization (RCL) on labor market outcomes using synthetic controls for the treated units. *Broadly-defined Agriculture* is the “Agriculture, Forestry, Fishing, and Hunting” (NAICS 11) industry. *Broadly-defined Retail* is the combination of “NAICS 446 Health and personal care stores,” “NAICS 452 General merchandise stores,” and “NAICS 453 Miscellaneous store retailers.” *P* values are calculated via a placebo test. Stars indicate standard significance levels: ^∗^10%, ^∗∗^5%, ^∗∗∗^1%

The difference in results between Colorado and Washington is potentially driven by the vertical integration requirement in Colorado and the vertical dis-integration requirement in Washington. In particular, firms in Colorado may classify themselves completely as cannabis retailers, as opposed to cannabis producers. While it is unlikely that these firms would compete with other agriculture firms for labor (and indeed even if firms are classified in this way, we see no effect on agricultural wages in Tables 1 and 2), it is possible that firms organized in this way have an effect on wages paid in the retail sector. We thus repeat the analysis once more for firms in related NAICS retail categories 446, 452, and 453. The results are reported in Table 2 under the heading “Broadly-defined Retail.” As expected, the estimates are attenuated from the more narrowly defined category. We find limited evidence to support the hypothesis that weekly per-worker wages increased in Colorado (the point estimate of a 1.5% increase is significant at the 10% level) and no evidence to support such a hypothesis in Washington.

### Robustness

In Table 3, we explore three alternative specifications, focusing on our primary outcome of average weekly wages per worker. In Column (2), we include only states with medical cannabis systems in our donor pool. In Column (3), we include only states with full prohibition of cannabis throughout our study period in our donor pool; the small number of states in this category limits the available inference. In Column (4), we follow the suggestion of Ferman and Pinto (2021) and repeat the analysis in levels while demeaning the outcomes. We do find potential evidence of a small increase in wages per worker in Washington in the broad retail category, though in context of the remainder of our estimates this is likely spurious.

**Table 3:** Results from alternative specifications of weekly wages per worker

	(1)	(2)	(3)	(4)
	Baseline	Med. cannabis controls only	Illegal controls only	In levels, demeaned
*Colorado*				
Narrow Agriculture	− 0.007	0.094*	0.077	14.22*
	[0.576]	[0.069]	[0.2]	[0.091]
Narrow Retail	0.050	− 0.094	− 0.044	-42.62
	[0.212]	[0.897]	[0.8]	[0.879]
Broad Agriculture	0.007	− 0.010	0.055	− 27.22
	[0.242]	[0.548]	[0.4]	[0.697]
Broad Retail	0.015*	− 0.033	− 0.063	− 291.63
	[0.061]	[0.586]	[0.4]	[0.818]
Washington				
Narrow Agriculture	− 0.013*	0.748*	0.695	397.8*
	[0.091]	[0.069]	[0.4]	[0.091]
Narrow Retail	0.059	0.158	0.198	− 178.2
	[0.606]	[0.103]	[0.2]	[0.121]
Broad Agriculture	− 0.154	− 0.050	− 0.020	− 1200.23
	[0.667]	[0.419]	[0.6]	[0.697]
Broad Retail	0.014	0.025	0.012	52.10**
	[0.333]	[0.103]	[0.4]	[0.030]

Notes: Narrow agriculture is NAICS 1114, narrow retail is NAICS 453998, broad agriculture is NAICS 11, broad retail is NAICS 446, 452, and 453. *P* values in brackets are calculated via a placebo test. Column (1) repeats results from Tables 1 and 2. In Column (2), we restrict the set of potential donor states to those with medical cannabis regimes. In Column (3), we restrict the set of potential donor states to those with full prohibition of cannabis throughout our study period. In Column (4), we use the level of average wages per worker per week (as opposed to the log wage) and demean the outcomes. Stars indicate significance levels: *10%, **5%, ***1

## Conclusion

Over the past decade, US voters have undergone a rapid shift towards supporting the legalization of cannabis in some form and policy has changed to follow this support. These changes, however, have not come without frictions generated by broad society-wide concerns about (among other issues) public health and safety (Hall and Lynskey 2016; Kilmer 2019), educational outcomes (van Ours and Williams 2015), and interactions with other substances (Miller and Seo 2021). Other frictions have been caused by more immediate financial concerns: agricultural firms in areas with legal cannabis production have expressed concerns about upward wage pressures leading to reduced international competitiveness and domestic agricultural output. Indeed, Bampasidou and Salassi (2019) identify a number of instances of labor shortages in particular US agricultural industries and regions around the time of the first successful cannabis legalization campaigns. At the same time, supporters of legalization have pointed to substantial employment within the nascent industry as a sign of success. Taken together, it is natural to suggest that cannabis legalization may be contributing to a highly competitive labor market from the perspective of agricultural employers.

We investigate the relationship between cannabis legalization and labor market outcomes across both the agricultural and retail sectors. Using a synthetic control approach paired with machine learning techniques including LASSO to select appropriate covariates on which to generate synthetic control units and CART for chained imputation of missing values, we ask whether equilibrium wages increased after legalization in Washington and Colorado, the first states to legalize. We find limited evidence to support this assertion; while the number of workers in the relevant sectors increased following the entry of cannabis producers and retailers, the wage per worker remained effectively constant.

Our results indicate that cannabis is not likely to be responsible for the broader changes in the agricultural or retail labor markets experienced during our study period. Indeed, others have pointed to changes in immigration policy including an increase in the intensity of enforcement (Escalante and Luo 2017) and frictions in the H-2A guest worker program (Luckstead and Devadoss 2019) as key contributing factors to changes in agricultural labor markets. On the retail side, aggregation in brick-and-mortar retailers (Neumark et al. 2008) and the increase in online shopping (Bram and Gorton 2017) have been identified as key drivers of changes in retail employment outcomes. Relative to these broader labor market trends, cannabis legalization may well be the proverbial “drop in the bucket.” At the same time, results from studies of MCLs suggest that increasing cannabis access may increase labor supply, though results from RCLs to this point have been mixed. If RCLs do increase labor supply, our null result could be explained by offsetting changes on the demand and supply side of the labor market. It is also possible that our results could be explained by the conversion of illegal production to legal production with minimal changes in the labor force (i.e., those who were engaged in illegal production became those employed by legal producers). More generally, if cannabis employment is particularly attractive to individuals who were not previously engaged in the labor market (including those who were unemployed or self-employed), our null result may well be expected.

These results are subject to a number of limitations which may be addressed by future work. While we have focused on the labor market motivated by anecdotal reports and popular press accounts, it is possible that the entry of adult-use cannabis firms may affect incumbent firms in the agricultural and retail sectors through other channels, such as competition for desirable real estate or within the product market. Our work is necessarily limited to a relatively short post-legalization period, and as cannabis production continues to grow, it is possible that other agricultural and retail firms may face competition from cannabis firms that differs from past experience. While many states have adopted regulatory frameworks similar to either Colorado’s or Washington’s, the details vary widely across dimensions including the number of licensed establishments, tax rates and licensing fees, quantity and potency limits, and out-of-state investment rules, among others (Hansen et al. 2021a). These differences may affect the cannabis industry’s aggregate demand for labor across states and therefore the experience of agricultural and retail incumbents. Indeed, both Colorado and Washington allow counties and municipalities to ban entry by cannabis firms, and so there may be within-state heterogeneity. Finally, both Colorado and Washington had existing medical cannabis systems before opening their recreational markets. Our results therefore speak only to the incremental effect of recreational legalization; a state moving from full prohibition to a fully-legal regime may experience a larger effect.

Our study may give policymakers currently considering cannabis liberalization some indication that such a policy change is unlikely to significantly increase wage bills for existing retailers and agricultural firms in the short term. Indeed, legalization is likely to improve labor market outcomes for job-seekers, if only by slightly increasing demand for labor—though long-term cannabis use may affect labor market outcomes at the individual level (Sabia and Nguyen 2018).

## Appendix A: Additional tables and figures

**Table 4:** Synthetic control weights assigned to each state for narrowly-defined agriculture labor market outcomes

	Log number of establishments	Log number of workers	Log real total quarterly wage	Log real average weekly wage per worker
*Colorado*				
Arizona	0.00	0.00	0.00	0.04
Georgia	0.19	0.00	0.38	0.00
Hawaii	0.00	0.25	0.00	0.00
Maryland	0.00	0.00	0.00	0.33
Minnesota	0.00	0.00	0.00	0.25
Montana	0.00	0.22	0.20	0.11
New Hampshire	0.19	0.00	0.00	0.00
South Carolina	0.00	0.00	0.00	0.23
Texas	0.45	0.53	0.41	0.04
Vermont	0.17	0.00	0.00	0.00
*Washington*				
Arizona	0.00	0.03	0.11	0.00
Connecticut	0.00	0.02	0.00	0.07
Florida	0.10	0.04	0.00	0.00
Georgia	0.00	0.00	0.00	0.37
Hawaii	0.00	0.05	0.01	0.07
Illinois	0.08	0.00	0.00	0.00
Kentucky	0.00	0.00	0.08	0.00
Michigan	0.54	0.40	0.05	0.00
Minnesota	0.28	0.37	0.00	0.00
Montana	0.00	0.00	0.00	0.14
South Dakota	0.00	0.00	0.00	0.15
Texas	0.00	0.08	0.66	0.19
West Virginia	0.00	0.00	0.09	0.00

Notes: The table provides the weights assigned to states for the synthetic controls used to estimate the “narrowly-defined agriculture” models in Table 1. All states except those which legalized cannabis during our study period and those bordering either Washington or Colorado were included in the pool of potential control units. Only states which received positive weight for at least one outcome are included in the table

**Table 5:** Synthetic control weights assigned to each state for narrowly-defined retail labor market outcomes

	Log number of establishments	Log number of workers	Log real total quarterly wage	Log real average weekly wage per worker
*Colorado*				
Georgia	0.07	0.11	0.19	0.00
Iowa	0.41	0.26	0.18	0.00
Kentucky	0.10	0.00	0.01	0.33
Louisiana	0.20	0.28	0.21	0.00
Minnesota	0.00	0.28	0.18	0.00
Mississippi	0.00	0.00	0.00	0.11
Missouri	0.00	0.00	0.01	0.00
New Hampshire	0.00	0.00	0.00	0.14
Pennsylvania	0.00	0.00	0.00	0.22
South Dakota	0.00	0.00	0.00	0.19
Texas	0.22	0.06	0.05	0.00
Wisconsin	0.00	0.00	0.18	0.00
*Washington*				
Connecticut	0.00	0.27	0.31	0.00
Illinois	0.00	0.38	0.36	0.00
Iowa	0.35	0.00	0.00	0.21
Michigan	0.33	0.04	0.05	0.07
Mississippi	0.05	0.00	0.00	0.00
New York	0.00	0.00	0.02	0.00
North Carolina	0.12	0.18	0.00	0.53
Pennsylvania	0.15	0.00	0.00	0.08
South Carolina	0.00	0.13	0.26	0.11

Notes: The table provides the weights assigned to states for the synthetic controls used to estimate the “narrowly-defined retail” models in Table 1. All states except those which legalized cannabis during our study period and those bordering either Washington or Colorado were included in the pool of potential control units. Only states which received positive weight for at least one outcome are included in the table

**Table 6:** Synthetic control weights assigned to each state for broadly-defined agriculture labor market outcomes

	Log number of establishments	Log number of workers	Log real total quarterly wage	Log real average weekly wage per worker
*Colorado*				
Arizona	0.19	0.26	0.12	0.00
Georgia	0.52	0.00	0.10	0.02
Hawaii	0.01	0.02	0.01	0.00
Kentucky	0.00	0.00	0.00	0.16
Minnesota	0.00	0.24	0.10	0.43
Montana	0.07	0.27	0.08	0.00
New Hampshire	0.00	0.05	0.22	0.00
South Dakota	0.21	0.00	0.00	0.00
Texas	0.00	0.15	0.29	0.02
Virginia	0.00	0.00	0.08	0.37
*Washington*				
Connecticut	0.00	0.00	0.00	0.07
Florida	0.00	0.86	0.74	0.18
Michigan	0.00	0.00	0.00	0.69
Minnesota	0.00	0.00	0.00	0.05
Montana	0.07	0.00	0.00	0.00
Texas	0.93	0.12	0.24	0.00

Notes: The table provides the weights assigned to states for the synthetic controls used to estimate the “broadly-defined agriculture” models in Table 2. All states except those which legalized cannabis during our study period and those bordering either Washington or Colorado were included in the pool of potential control units; Alaska and California were added to the pool for Washington due to the similarity in their agriculture, forestry, and fishing industries. Only states which received positive weight for at least one outcome are included in the table

**Table 7:** Synthetic control weights assigned to each state for broadly-defined retail labor market outcomes

	Log number of establishments	Log number of workers	Log real total quarterly wage	Log real average weekly wage per worker
*Colorado*				
Alabama	0.02	0.01	0.02	0.02
Arizona	0.02	0.18	0.11	0.02
Arkansas	0.02	0.01	0.03	0.03
Connecticut	0.23	0.00	0.00	0.01
Florida	0.00	0.00	0.00	0.01
Georgia	0.02	0.00	0.01	0.02
Hawaii	0.04	0.00	0.01	0.00
Illinois	0.01	0.00	0.01	0.03
Indiana	0.02	0.00	0.01	0.03
Iowa	0.02	0.01	0.02	0.05
Kentucky	0.02	0.01	0.01	0.02
Louisiana	0.03	0.01	0.02	0.05
Maryland	0.02	0.00	0.00	0.01
Michigan	0.01	0.00	0.01	0.02
Minnesota	0.02	0.01	0.03	0.07
Mississippi	0.02	0.01	0.03	0.03
Missouri	0.02	0.01	0.02	0.03
Montana	0.03	0.35	0.29	0.03
New Hampshire	0.03	0.00	0.01	0.03
New Jersey	0.07	0.00	0.00	0.01
New York	0.12	0.00	0.00	0.00
North Carolina	0.01	0.00	0.01	0.02
Ohio	0.01	0.00	0.00	0.02
Pennsylvania	0.01	0.00	0.00	0.02
South Carolina	0.02	0.00	0.01	0.02
South Dakota	0.03	0.04	0.04	0.06
Tennessee	0.02	0.00	0.01	0.01
Texas	0.01	0.34	0.28	0.19
Vermont	0.03	0.00	0.00	0.02
Virginia	0.02	0.00	0.01	0.04
West Virginia	0.02	0.01	0.01	0.03
Wisconsin	0.02	0.01	0.01	0.04
*Washington*				
Alabama	0.00	0.00	0.00	0.01
Connecticut	0.38	0.00	0.00	0.00
Georgia	0.00	0.00	0.00	0.01
Hawaii	0.00	0.00	0.00	0.09
Illinois	0.28	0.00	0.00	0.01
Iowa	0.00	0.24	0.01	0.00
Michigan	0.00	0.00	0.00	0.01
Minnesota	0.00	0.00	0.00	0.01
Missouri	0.00	0.00	0.00	0.01
New York	0.02	0.01	0.00	0.49
North Carolina	0.01	0.31	0.01	0.01
Pennsylvania	0.01	0.00	0.00	0.00
South Carolina	0.27	0.22	0.23	0.00
Texas	0.00	0.00	0.00	0.31
Vermont	0.00	0.00	0.00	0.01
Virginia	0.00	0.21	0.74	0.00

Notes: The table provides the weights assigned to states for the synthetic controls used to estimate the “broadly-defined retail” models in Table 1. All states except those which legalized cannabis during our study period and those bordering either Washington or Colorado were included in the pool of potential control units. Only states which received positive weight for at least one outcome are included in the table

**Fig. 7:**
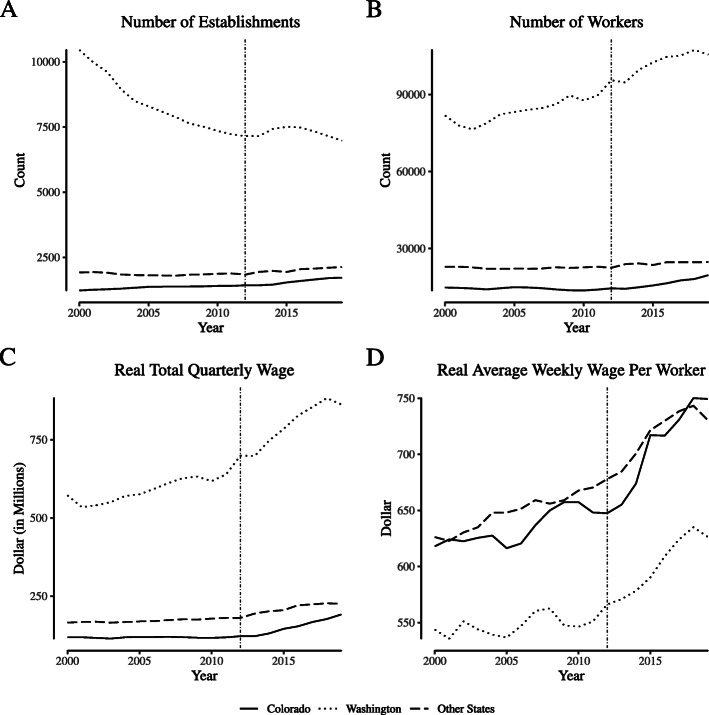
Employment and wages for “broadly defined” agricultural firms. Notes: Data come from the Quarterly Census of Employment and Wages. We define “broadly defined” agricultural firms as those within NAICS 11 (“Agriculture, Forestry, Fishing, and Hunting”)

**Fig. 8:**
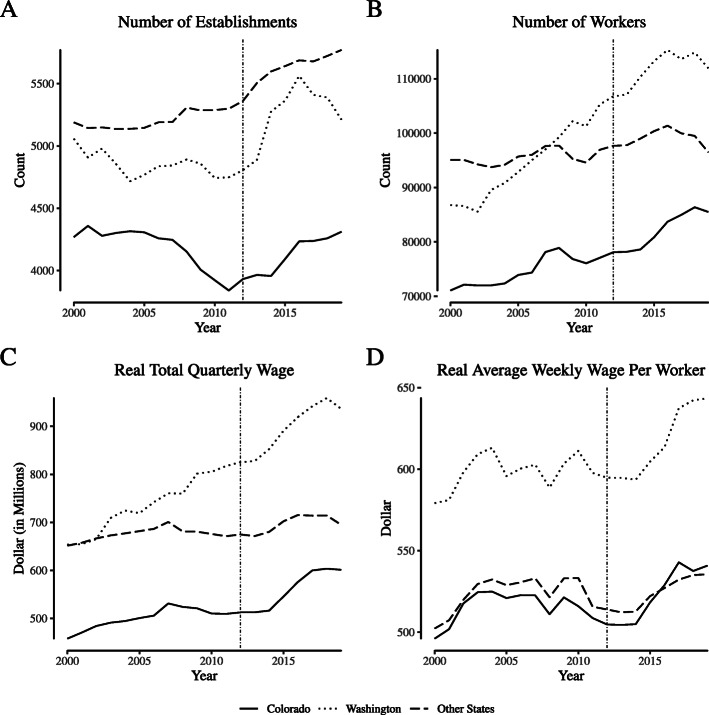
Employment and wages for “broadly defined” retail firms. Notes: Data come from the Quarterly Census of Employment and Wages. We define “broadly defined” retail firms as those within NAICS 446, 452, and 453 (“Health and personal care stores,” “General merchandise stores,” and “Miscellaneous stores,” respectively)

**Fig. 9:**
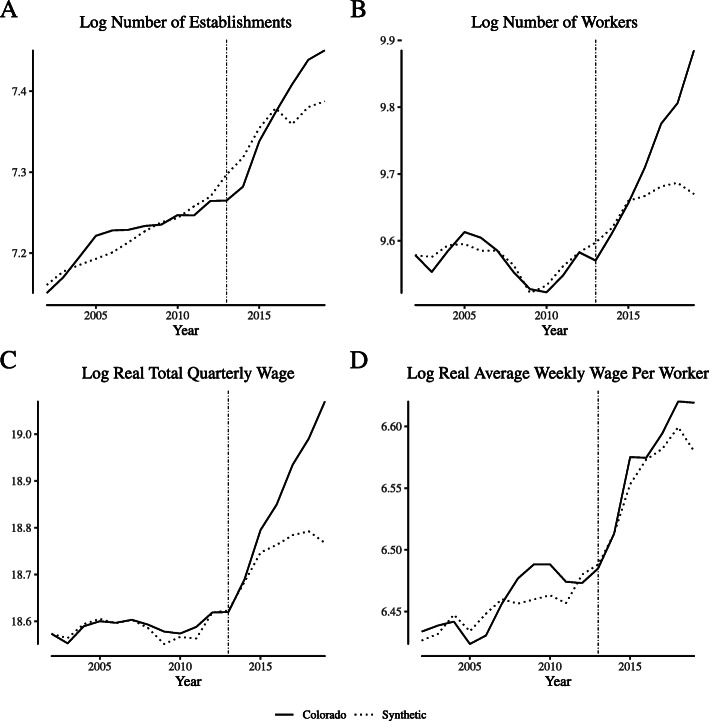
Comparing broadly-defined agriculture labor market outcomes in Colorado and its synthetic control. Notes: This figure depicts wage and employment outcomes for “broadly defined” agricultural firms for Colorado and its synthetic control. We define “broadly defined” agricultural firms as those within NAICS 11 (“Agriculture, Forestry, Fishing, and Hunting”)

**Fig. 10:**
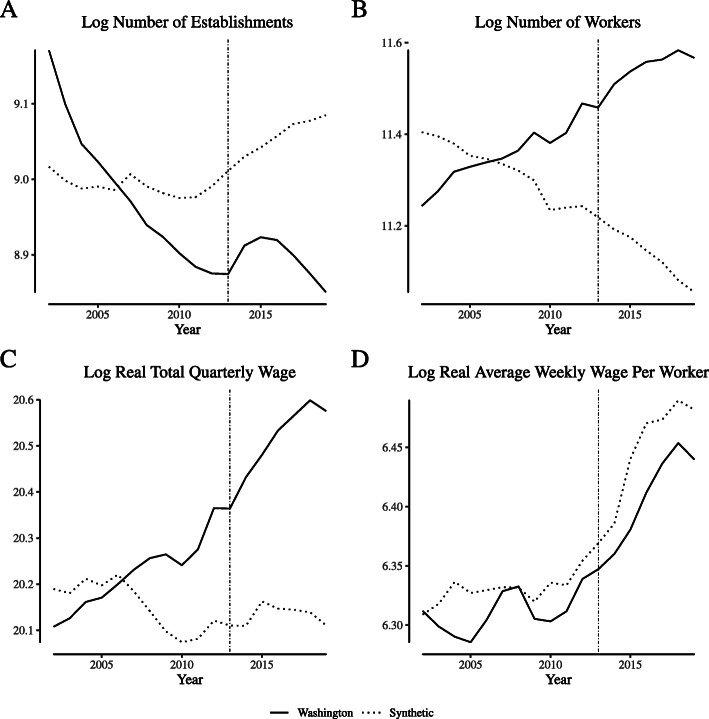
Comparing broadly-defined agriculture labor market outcomes in Washington and its synthetic control. Notes: This figure depicts wage and employment outcomes for “broadly defined” agricultural firms for Washington and its synthetic control. We define “broadly defined” agricultural firms as those within NAICS 11 (“Agriculture, Forestry, Fishing, and Hunting”)

**Fig. 11:**
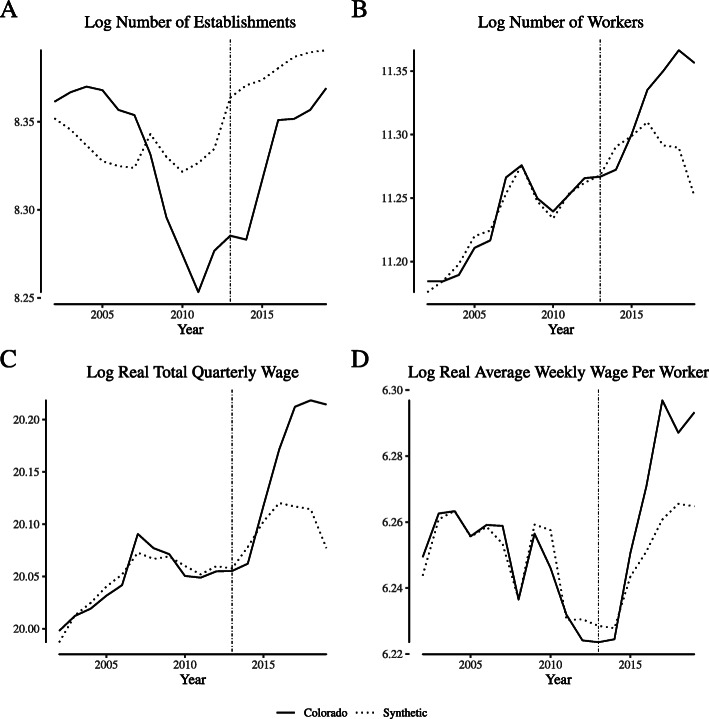
Comparing broadly-defined retailer labor market outcomes in Colorado and its synthetic control. Notes: This figure depicts wage and employment outcomes for “broadly defined” retail firms for Colorado and its synthetic control. We define “broadly defined” retail firms as those within NAICS 446, 452, and 453 (“Health and personal care stores,” “General merchandise stores,” and “Miscellaneous stores,” respectively)

**Fig. 12:**
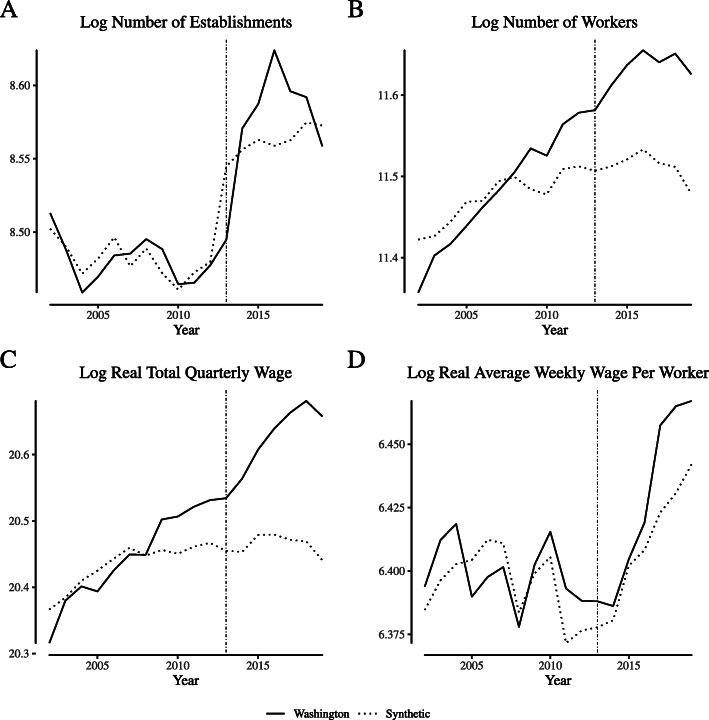
Comparing broadly-defined retailer labor market outcomes in Washington and its synthetic control. Notes: This figure depicts wage and employment outcomes for “broadly defined” retail firms for Washington and its synthetic control. We define “broadly defined” retail firms as those within NAICS 446, 452, and 453 (“Health and personal care stores,” “General merchandise stores,” and “Miscellaneous stores,” respectively)

**Fig. 13:**
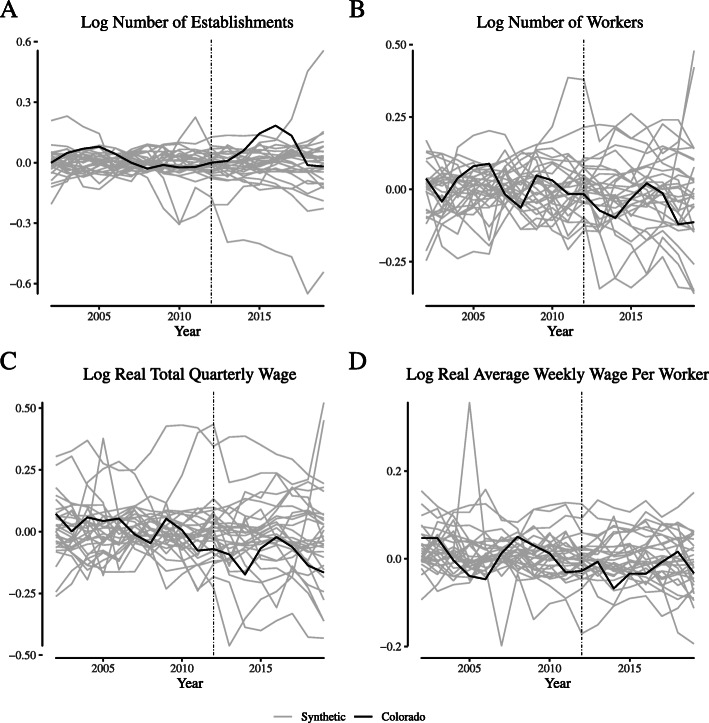
Placebo tests for narrowly-defined agriculture labor market outcomes in Colorado. Notes: This figure depicts the placebo tests for “narrowly defined” agricultural firms for Colorado. We define “narrowly defined” agricultural firms as those within the “Greenhouse and Nursery Production” (NAICS 1114) industry

**Fig. 14:**
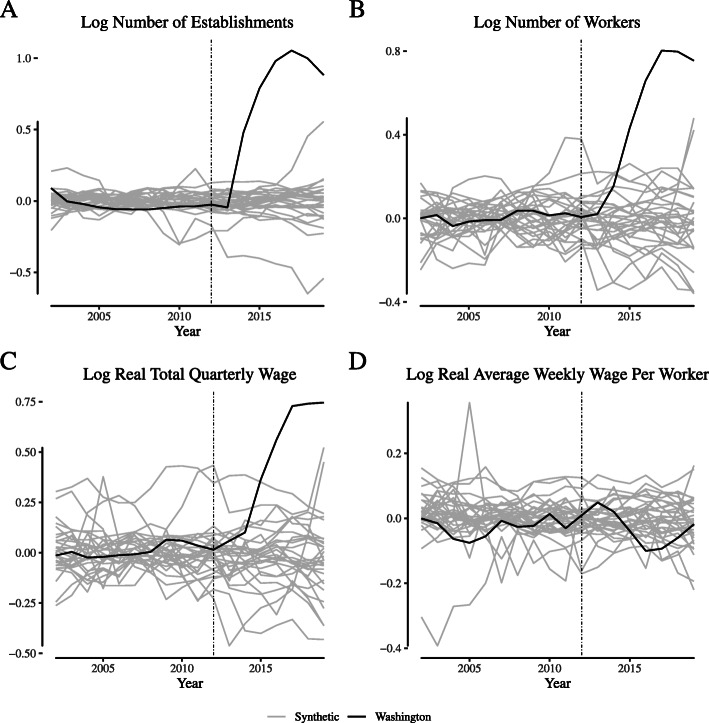
Placebo tests for narrowly-defined agriculture labor market outcomes in Washington. Notes: This figure depicts the placebo tests for “narrowly defined” agricultural firms for Washington. We define “narrowly defined” agricultural firms as those within the “Greenhouse and Nursery Production” (NAICS 1114) industry

**Fig. 15:**
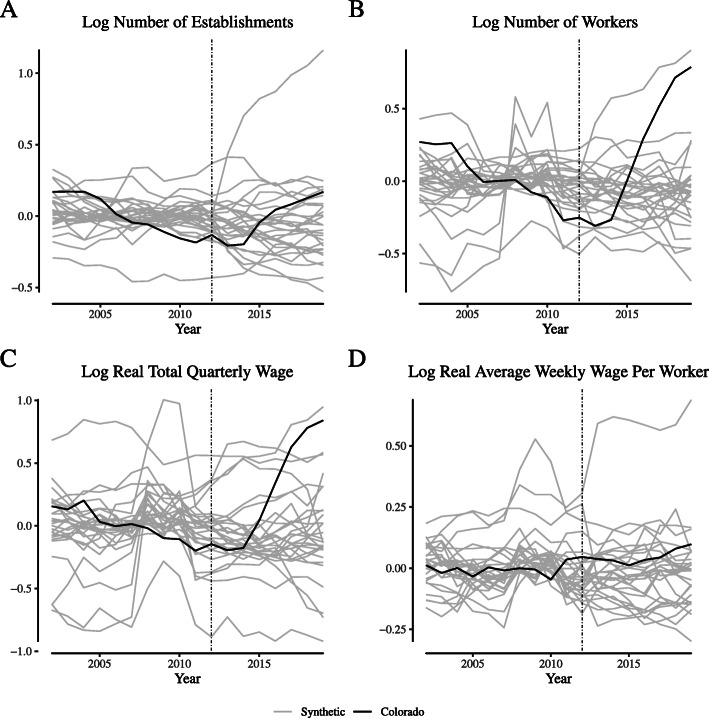
Placebo tests for narrowly-defined retailer labor market outcomes in Colorado. Notes: This figure depicts the placebo tests for wage and employment outcomes for “narrowly defined” retail firms for Colorado. We define “narrowly defined” retail firms as those within the “Store retailers not specified elsewhere” category (NAICS 453998)

**Fig. 16:**
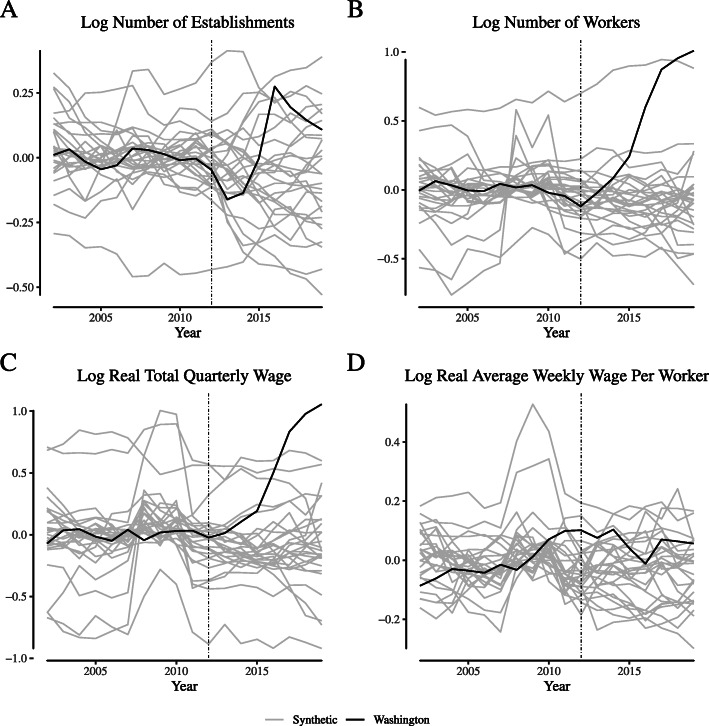
Placebo tests for narrowly-defined retailer labor market outcomes in Washington. Notes: This figure depicts the placebo tests for wage and employment outcomes for “narrowly defined” retail firms for Washington. We define “narrowly defined” retail firms as those within the “Store retailers not specified elsewhere” category (NAICS 453998)

**Fig. 17:**
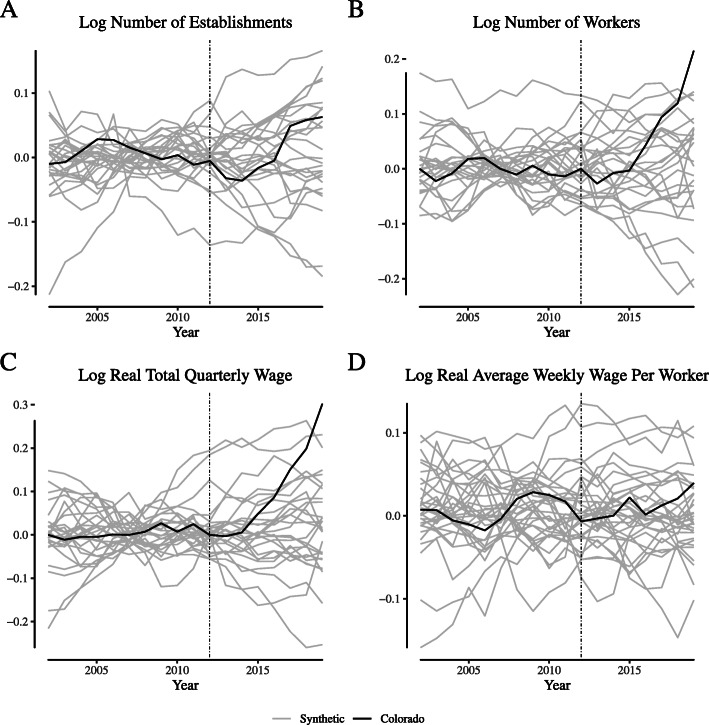
Placebo tests for broadly-defined agriculture labor market outcomes in Colorado. Notes: This figure depicts the placebo tests for “broadly defined” agricultural firms for Colorado. We define “broadly defined” agricultural firms as those within NAICS 11 (“Agriculture, Forestry, Fishing, and Hunting”)

**Fig. 18:**
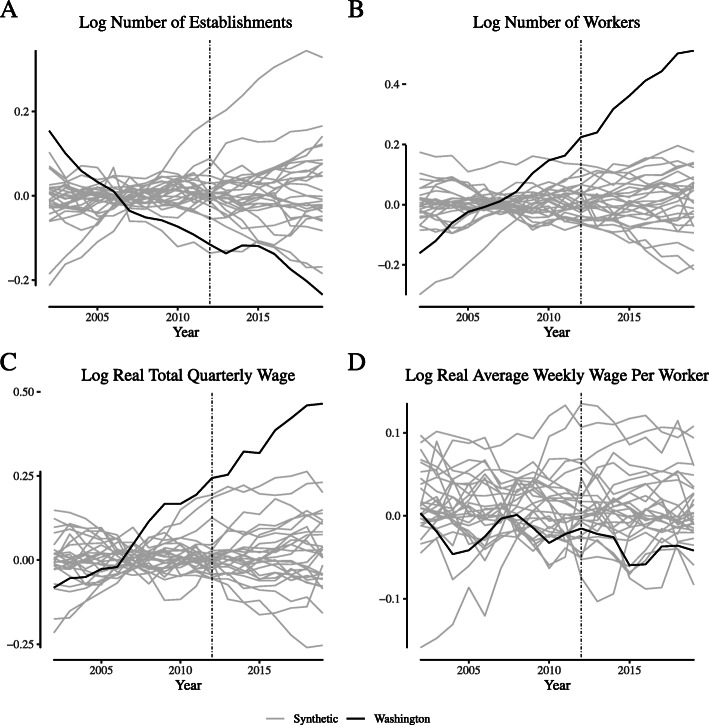
Placebo tests for broadly-defined agriculture labor market outcomes in Washington. Notes: This figure depicts the placebo tests for “broadly defined” agricultural firms for Washington. We define “broadly defined” agricultural firms as those within NAICS 11 (“Agriculture, Forestry, Fishing, and Hunting”)

**Fig. 19:**
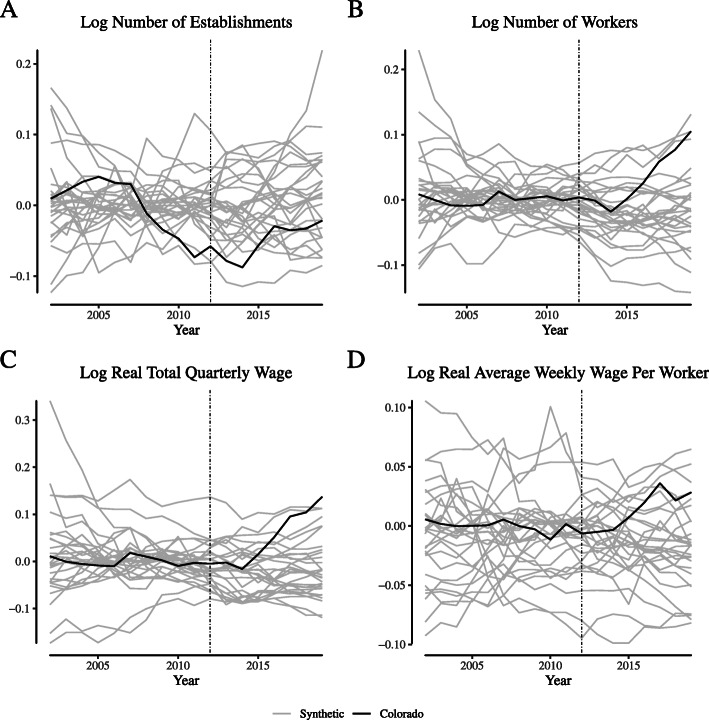
Placebo tests for broadly-defined retailer labor market outcomes in Colorado. Notes: This figure depicts the placebo tests for wage and employment outcomes for “broadly defined” retail firms for Colorado. We define “broadly defined” retail firms as those within NAICS 446, 452, and 453 (“Health and personal care stores,” “General merchandise stores,” and “Miscellaneous stores,” respectively)

**Fig. 20:**
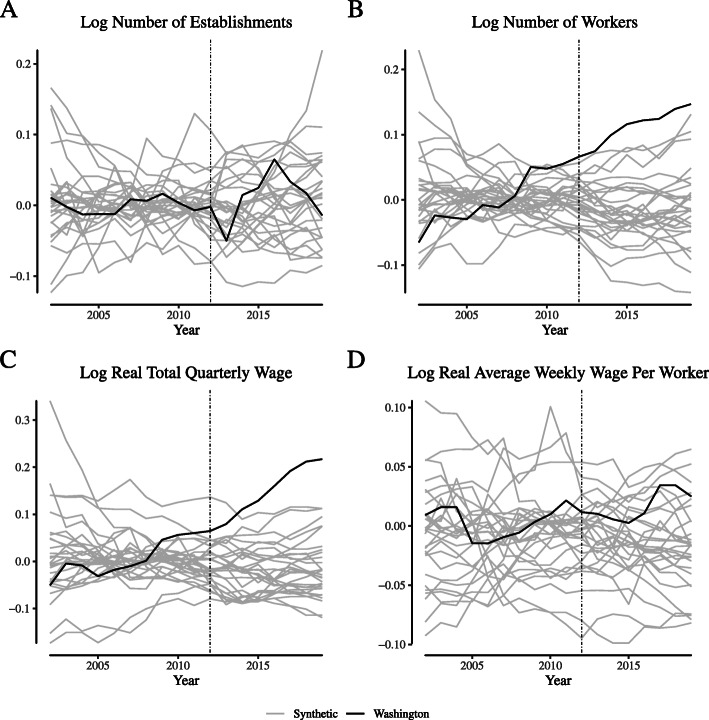
Placebo tests for broadly-defined retailer labor market outcomes in Washington. Notes: This figure depicts the placebo tests for wage and employment outcomes for “broadly defined” retail firms for Washington. We define “broadly defined” retail firms as those within NAICS 446, 452, and 453 (“Health and personal care stores,” “General merchandise stores,” and “Miscellaneous stores,” respectively)

## Appendix B: Tables of covariate balance

**Table 8:** CO broadly-defined agriculture average weekly wage per worker

	Treated	Synthetic	Sample mean
Lagged outcome	6.46	6.45	6.45
Barley for grain (acres)	65,547.33	64,022.64	41,592.84
Land in orchards (acres)	6444.00	18,069.70	51,262.40
Snap beans harvested for sale, harvested (acres)	590.67	4442.38	8048.64
Fruits and nuts, cherries, tart, total acres (acres)	159.67	28.15	2208.25
Fruits and nuts, pears, all, total acres (acres)	313.67	128.15	227.10
Comm. soil conds. (thousands of treated acres)	4130.86	8059.39	5357.77
Resident population 65 years and over (percent)	10.29	11.95	13.03
Savings institutions - total deposits (thousands)	1210.38	1377.93	2692.87
Civilian labor force unemployment rate (percent)	5.36	4.93	5.53
Federal Government expenditure-grants (millions)	6.04	8.5	10.89
Federal Government insurance (millions)	3.89	12.04	25.58
Resident population: Black alone (percent)	4.25	11.00	13.53
Resident population: two or more races (percent)	1.79	1.42	1.71
Resident population: Hispanic or Latino Origin (percent)	17.58	5.92	7.41
Resident population: total females (percent)	49.70	50.64	50.93
Social security: retired workers-benefit recipients (thousands)	386.55	612.67	716.18
Corn Grain Production (dollar, millions)	492.99	1678.67	1045.22
Hay production (dollar, millions)	493.11	412.94	260.35
Farm operations (acres, millions)	62.65	39.43	34.27
Labor hired wage (per hour)	8.50	8.64	11.20
Rent cash cropland expense (acres)	60.00	77.05	75.20
Vegetable totals (dollars, millions)	110.31	56.15	140.21
Wheat production (dollars, millions)	352.67	233.34	138.08

**Table 9:** CO broadly-defined agriculture total quarterly wages

	Treated	Synthetic	Sample mean
Lagged outcome	18.59	18.58	18.37
Barley for grain (acres)	65,547.33	87,647.33	41,592.84
Land in orchards (acres)	6444.00	86,064.13	51,262.40
Snap beans harvested for sale, harvested (acres)	590.67	4755.89	8048.64
Fruits and nuts, cherries, tart, total acres (acres)	159.67	105.70	2208.25
Fruits and nuts, pears, all, total acres (acres)	313.67	247.04	227.10
Comm. soil conds. (thousands of treated acres)	4130.86	8048.08	5357.77
Resident population 65 years and over (percent)	10.29	11.79	13.03
Savings institutions - total deposits (thousands)	1210.38	2651.68	2692.87
Civilian labor force unemployment rate (percent)	5.36	5.10	5.53
Federal Government expenditure-grants (millions)	6.04	13.57	10.89
Federal Government insurance (millions)	3.89	36.76	25.58
Resident population: Black alone (percent)	4.25	9.76	13.53
Resident population: two or more races (percent)	1.79	1.44	1.71
Resident population: Hispanic or Latino origin (percent)	17.58	15.23	7.41
Resident population: total females (percent)	49.70	50.44	50.93
Social security: retired workers-benefit recipients (thousands)	386.55	825.32	716.18
Corn Grain Production (dollar, millions)	492.99	728.86	1045.22
Hay production (dollar, millions)	493.11	439.95	260.35
Farm operations (acres, millions)	62.65	99.11	34.27
Labor hired wage (per hour)	8.50	10.15	11.20
Rent cash cropland expense (acres)	60.00	65.61	75.20
Vegetable totals (dollars, millions)	110.31	194.72	140.21
Wheat production (dollars, millions)	352.67	216.10	138.08

**Table 10:** CO broadly-defined agriculture average employment

	Treated	Synthetic	Sample mean
Lagged outcome	9.57	9.57	9.36
Barley for grain (acres)	65,547.33	258,337.20	41,592.84
Land in orchards (acres)	6444.00	47,636.03	51,262.40
Snap beans harvested for sale, harvested (acres)	590.67	2589.34	8048.64
Fruits and nuts, cherries, tart, total acres (acres)	159.67	89.21	2208.25
Fruits and nuts, pears, all, total acres (acres)	313.67	118.67	227.10
Comm. soil conds. (thousands of treated acres)	4130.86	8445.85	5357.77
Resident population 65 years and over (percent)	10.29	12.61	13.03
Savings institutions - total deposits (thousands)	1210.38	1339.26	2692.87
Civilian labor force unemployment rate (percent)	5.36	5.15	5.53
Federal Government expenditure-grants (millions)	6.04	9.72	10.89
Federal Government insurance (millions)	3.89	19.85	25.58
Resident population: Black alone (percent)	4.25	5.00	13.53
Resident population: two or more races (percent)	1.79	1.70	1.71
Resident population: Hispanic or Latino origin (percent)	17.58	14.14	7.41
Resident population: total females (percent)	49.70	50.23	50.93
Social security: retired workers-benefit recipients (thousands)	386.55	600.74	716.18
Corn Grain Production (dollar, millions)	492.99	1036.97	1045.22
Hay production (dollar, millions)	493.11	451.59	260.35
Farm operations (acres, millions)	62.65	92.69	34.27
Labor hired wage (per hour)	8.50	12.82	11.20
Rent cash cropland expense (acres)	60.00	71.83	75.20
Vegetable totals (dollars, millions)	110.31	246.95	140.21
Wheat production (dollars, millions)	352.67	396.12	138.08

**Table 11:** CO broadly-defined agriculture number of establishments

	Treated	Synthetic	Sample mean
Lagged outcome	7.22	7.21	7.15
Barley for grain (acres)	65,547.33	72,538.36	41,592.84
Land in orchards (acres)	6444.00	81,145.72	51,262.40
Snap beans harvested for sale, harvested (acres)	590.67	7659.43	8048.64
Fruits and nuts, cherries, tart, total acres (acres)	159.67	13.79	2208.25
Fruits and nuts, pears, all, total acres (acres)	313.67	161.75	227.10
Comm. soil conds. (thousands of treated acres)	4130.86	4692.31	5357.77
Resident population 65 years and over (percent)	10.29	11.78	13.03
Savings institutions - total deposits (thousands)	1210.38	576.47	2692.87
Civilian labor force unemployment rate (percent)	5.36	5.16	5.53
Federal Government expenditure-grants (millions)	6.04	8.54	10.89
Federal Government insurance (millions)	3.89	10.31	25.58
Resident population: Black alone (percent)	4.25	16.11	13.53
Resident population: two or more races (percent)	1.79	1.47	1.71
Resident population: Hispanic or Latino origin (percent)	17.58	9.66	7.41
Resident population: total females (percent)	49.70	50.52	50.93
Social security: retired workers-benefit recipients (thousands)	386.55	533.48	716.18
Corn Grain Production (dollar, millions)	492.99	427.44	1045.22
Hay production (dollar, millions)	493.11	253.37	260.35
Farm operations (acres, millions)	62.65	42.90	34.27
Labor hired wage (per hour)	8.50	11.43	11.20
Rent cash cropland expense (acres)	60.00	70.24	75.20
Vegetable totals (dollars, millions)	110.31	282.20	140.21
Wheat production (dollars, millions)	352.67	214.24	138.08

**Table 12:** WA broadly-defined agriculture average weekly wage per worker

	Treated	Synthetic	Sample mean
Lagged outcome	6.31	6.33	6.45
Barley for grain (acres)	245,385.00	17,117.25	41,592.84
Land in orchards (acres)	308,608.00	209,756.76	51,262.40
Snap beans harvested for sale, harvested (acres)	3418.67	19,386.12	8048.64
Fruits and nuts, cherries, tart, total acres (acres)	1976.33	30,113.96	2208.25
Fruits and nuts, pears, all, total acres (acres)	26,240.67	751.24	227.10
Comm. soil conds. (thousands of treated acres)	3959.26	5087.90	5357.77
Resident population 65 years and over (percent)	11.55	13.50	13.03
Savings institutions - total deposits (thousands)	3693.15	4339.17	2692.87
Civilian labor force unemployment rate (percent)	6.50	6.98	5.53
Federal Government expenditure-grants (millions)	9.92	14.84	10.89
Federal Government insurance (millions)	7.33	69.71	25.58
Resident population: Black alone (percent)	4.45	13.37	13.53
Resident population: two or more races (percent)	2.78	1.40	1.71
Resident population: Hispanic or Latino origin (percent)	9.38	7.01	7.41
Resident population: total females (percent)	50.23	50.91	50.93
Social security: retired workers-benefit recipients (thousands)	622.15	1242.64	716.18
Corn Grain Production (dollar, millions)	78.87	911.96	1045.22
Hay production (dollar, millions)	445.95	247.26	260.35
Farm operations (acres, millions)	30.02	20.39	34.27
Labor hired wage (per hour)	9.50	14.85	11.20
Rent cash cropland expense (acres)	136.50	81.52	75.20
Vegetable totals (dollars, millions)	182.97	362.61	140.21
Wheat production (dollars, millions)	782.89	168.52	138.08

**Table 13:** WA broadly-defined agriculture total quarterly wages

	Treated	Synthetic	Sample mean
Lagged outcome	20.20	20.16	18.37
Barley for grain (acres)	245,385.00	8933.69	41,592.84
Land in orchards (acres)	308,608.00	586,488.64	51,262.40
Snap beans harvested for sale, harvested (acres)	3418.67	28,444.94	8048.64
Fruits and nuts, cherries, tart, total acres (acres)	1976.33	20,969.07	2208.25
Fruits and nuts, pears, all, total acres (acres)	26,240.67	236.82	227.10
Comm. soil conds. (thousands of treated acres)	3959.26	6550.59	5357.77
Resident population 65 years and over (percent)	11.55	15.42	13.03
Savings institutions - total deposits (thousands)	3693.15	6468.79	2692.87
Civilian labor force unemployment rate (percent)	6.50	5.76	5.53
Federal Government expenditure-grants (millions)	9.92	23.07	10.89
Federal Government insurance (millions)	7.33	296.41	25.58
Resident population: Black alone (percent)	4.45	14.89	13.53
Resident population: two or more races (percent)	2.78	1.28	1.71
Resident population: Hispanic or Latino origin (percent)	9.38	21.37	7.41
Resident population: total females (percent)	50.23	50.86	50.93
Social security: retired workers-benefit recipients (thousands)	622.15	2187.51	716.18
Corn Grain Production (dollar, millions)	78.87	214.88	1045.22
Hay production (dollar, millions)	445.95	301.58	260.35
Farm operations (acres, millions)	30.02	78.23	34.27
Labor hired wage (per hour)	9.50	32.52	11.20
Rent cash cropland expense (acres)	136.50	77.57	75.20
Vegetable totals (dollars, millions)	182.97	1073.11	140.21
Wheat production (dollars, millions)	782.89	65.42	138.08

**Table 14:** WA broadly-defined agriculture average employment

	Treated	Synthetic	Sample mean
Lagged outcome	11.34	11.33	9.36
Barley for grain (acres)	245,385.00	10,088.92	41,592.84
Land in orchards (acres)	308,608.00	642,996.75	51,262.40
Snap beans harvested for sale, harvested (acres)	3418.67	31,630.43	8048.64
Fruits and nuts, cherries, tart, total acres (acres)	1976.33	24,223.85	2208.25
Fruits and nuts, pears, all, total acres (acres)	26,240.67	171.85	227.10
Comm. soil conds. (thousands of treated acres)	3959.26	4600.35	5357.77
Resident population 65 years and over (percent)	11.55	16.18	13.03
Savings institutions - total deposits (thousands)	3693.15	6420.39	2692.87
Civilian labor force unemployment rate (percent)	6.50	5.75	5.53
Federal Government expenditure-grants (millions)	9.92	21.60	10.89
Federal Government insurance (millions)	7.33	325.02	25.58
Resident population: Black alone (percent)	4.45	15.18	13.53
Resident population: two or more races (percent)	2.78	1.29	1.71
Resident population: Hispanic or Latino origin (percent)	9.38	19.48	7.41
Resident population: total females (percent)	50.23	50.94	50.93
Social security: retired workers-benefit recipients (thousands)	622.15	2241.95	716.18
Corn Grain Production (dollar, millions)	78.87	134.56	1045.22
Hay production (dollar, millions)	445.95	191.14	260.35
Farm operations (acres, millions)	30.02	47.49	34.27
Labor hired wage (per hour)	9.50	36.43	11.20
Rent cash cropland expense (acres)	136.50	85.01	75.20
Vegetable totals (dollars, millions)	182.97	1198.46	140.21
Wheat production (dollars, millions)	782.89	34.54	138.08

**Table 15:** WA broadly-defined agriculture number of establishments

	Treated	Synthetic	Sample mean
Lagged outcome	9.00	8.99	7.15
Barley for grain (acres)	245,385.00	62,094.10	41,592.84
Land in orchards (acres)	308,608.00	199,298.35	51,262.40
Snap beans harvested for sale, harvested (acres)	3418.67	7564.68	8048.64
Fruits and nuts, cherries, tart, total acres (acres)	1976.33	292.81	2208.25
Fruits and nuts, pears, all, total acres (acres)	26,240.67	586.85	227.10
Comm. soil conds. (thousands of treated acres)	3959.26	17,524.58	5357.77
Resident population 65 years and over (percent)	11.55	10.91	13.03
Savings institutions - total deposits (thousands)	3693.15	6096.23	2692.87
Civilian labor force unemployment rate (percent)	6.50	5.72	5.53
Federal Government expenditure-grants (millions)	9.92	29.17	10.89
Federal Government insurance (millions)	7.33	100.50	25.58
Resident population: Black alone (percent)	4.45	12.36	13.53
Resident population: two or more races (percent)	2.78	1.25	1.71
Resident population: Hispanic or Latino origin (percent)	9.38	29.70	7.41
Resident population: total females (percent)	50.23	50.33	50.93
Social security: retired workers-benefit recipients (thousands)	622.15	1658.19	716.18
Corn Grain Production (dollar, millions)	78.87	718.13	1045.22
Hay production (dollar, millions)	445.95	926.67	260.35
Farm operations (acres, millions)	30.02	250.92	34.27
Labor hired wage (per hour)	9.50	7.59	11.20
Rent cash cropland expense (acres)	136.50	32.10	75.20
Vegetable totals (dollars, millions)	182.97	249.71	140.21
Wheat production (dollars, millions)	782.89	302.52	138.08

**Table 16:** CO narrowly-defined agriculture average weekly wage per worker

	Treated	Synthetic	Sample Mean
Lagged outcome	6.46	6.45	6.34
Barley for grain (acres)	65,547.33	120,968.85	41,592.84
Land in orchards (acres)	6444.00	19,911.85	51,262.40
Snap beans harvested for sale, harvested (acres)	590.67	4764.35	8048.64
Fruits and nuts, cherries, tart, total acres (acres)	159.67	50.67	2208.25
Fruits and nuts, pears, all, total acres (acres)	313.67	91.63	227.10
Comm. soil conds. (thousands of treated acres)	4130.86	7547.31	5357.77
Resident population 65 years and over (percent)	10.29	11.98	13.03
Savings institutions - total deposits (thousands)	1210.38	1257.63	2692.87
Civilian labor force unemployment rate (percent)	5.36	4.97	5.53
Federal Government expenditure-grants (millions)	6.04	8.89	10.89
Federal Government insurance (millions)	3.89	9.88	25.58
Resident population: Black alone (percent)	4.25	13.61	13.53
Resident population: two or more races (percent)	1.79	1.44	1.71
Resident population: Hispanic or Latino origin (percent)	17.58	7.24	7.41
Resident population: total females (percent)	49.70	50.75	50.93
Social security: retired workers-benefit recipients (thousands)	386.55	542.95	716.18
Corn Grain Production (dollar, millions)	492.99	1575.22	1045.22
Hay production (dollar, millions)	493.11	311.07	260.35
Farm operations (acres, millions)	62.65	41.48	34.27
Labor hired wage (per hour)	8.50	10.21	11.20
Rent cash cropland expense (acres)	60.00	81.00	75.20
Vegetable totals (dollars, millions)	110.31	111.04	140.21
Wheat production (dollars, millions)	352.67	268.18	138.08

**Table 17:** CO narrowly-defined agriculture total quarterly wages

	Treated	Synthetic	Sample mean
Lagged outcome	17.01	17.00	16.39
Barley for grain (acres)	65,547.33	155,321.91	41,592.84
Land in orchards (acres)	6444.00	140,953.22	51,262.40
Snap beans harvested for sale, harvested (acres)	590.67	9375.06	8048.64
Fruits and nuts, cherries, tart, total acres (acres)	159.67	133.44	2208.25
Fruits and nuts, pears, all, total acres (acres)	313.67	370.37	227.10
Comm. soil conds. (thousands of treated acres)	4130.86	9932.14	5357.77
Resident population 65 years and over (percent)	10.29	11.01	13.03
Savings institutions - total deposits (thousands)	1210.38	2910.26	2692.87
Civilian labor force unemployment rate (percent)	5.36	5.52	5.53
Federal Government expenditure-grants (millions)	6.04	17.65	10.89
Federal Government insurance (millions)	3.89	48.94	25.58
Resident population: Black alone (percent)	4.25	17.28	13.53
Resident population: two or more races (percent)	1.79	1.26	1.71
Resident population: Hispanic or Latino origin (percent)	17.58	15.83	7.41
Resident population: total females (percent)	49.70	50.51	50.93
Social security: retired workers-benefit recipients (thousands)	386.55	1026.54	716.18
Corn Grain Production (dollar, millions)	492.99	384.11	1045.22
Hay production (dollar, millions)	493.11	499.44	260.35
Farm operations (acres, millions)	62.65	133.07	34.27
Labor hired wage (per hour)	8.50	7.91	11.20
Rent cash cropland expense (acres)	60.00	46.64	75.20
Vegetable totals (dollars, millions)	110.31	233.26	140.21
Wheat production (dollars, millions)	352.67	291.41	138.08

**Table 18:** CO narrowly-defined agriculture average employment

	Treated	Synthetic	Sample mean
Lagged outcome	8.00	7.98	7.48
Barley for grain (acres)	65,547.33	201,800.53	41,592.84
Land in orchards (acres)	6444.00	132,432.44	51,262.40
Snap beans harvested for sale, harvested (acres)	590.67	7592.61	8048.64
Fruits and nuts, cherries, tart, total acres (acres)	159.67	155.41	2208.25
Fruits and nuts, pears, all, total acres (acres)	313.67	358.83	227.10
Comm. soil conds. (thousands of treated acres)	4130.86	10,667.28	5357.77
Resident population 65 years and over (percent)	10.29	11.41	13.03
Savings institutions - total deposits (thousands)	1210.38	3147.63	2692.87
Civilian labor force unemployment rate (percent)	5.36	5.39	5.53
Federal Government expenditure-grants (millions)	6.04	17.55	10.89
Federal Government insurance (millions)	3.89	52.10	25.58
Resident population: Black alone (percent)	4.25	14.02	13.53
Resident population: two or more races (percent)	1.79	1.98	1.71
Resident population: Hispanic or Latino origin (percent)	17.58	16.85	7.41
Resident population: total females (percent)	49.70	50.40	50.93
Social security: retired workers-benefit recipients (thousands)	386.55	1011.87	716.18
Corn Grain Production (dollar, millions)	492.99	435.23	1045.22
Hay production (dollar, millions)	493.11	553.07	260.35
Farm operations (acres, millions)	62.65	149.25	34.27
Labor hired wage (per hour)	8.50	9.84	11.20
Rent cash cropland expense (acres)	60.00	45.00	75.20
Vegetable totals (dollars, millions)	110.31	219.30	140.21
Wheat production (dollars, millions)	352.67	346.31	138.08

**Table 19:** CO narrowly-defined agriculture number of establishments

	Treated	Synthetic	Sample mean
Lagged outcome	5.05	5.03	4.82
Barley for grain (acres)	65,547.33	197,582.96	41,592.84
Land in orchards (acres)	6444.00	63,451.92	51,262.40
Snap beans harvested for sale, harvested (acres)	590.67	5331.53	8048.64
Fruits and nuts, cherries, tart, total acres (acres)	159.67	300.51	2208.25
Fruits and nuts, pears, all, total acres (acres)	313.67	194.82	227.10
Comm. soil conds. (thousands of treated acres)	4130.86	8550.48	5357.77
Resident population 65 years and over (percent)	10.29	11.99	13.03
Savings institutions - total deposits (thousands)	1210.38	1793.17	2692.87
Civilian labor force unemployment rate (percent)	5.36	4.97	5.53
Federal Government expenditure-grants (millions)	6.04	10.85	10.89
Federal Government insurance (millions)	3.89	24.73	25.58
Resident population: Black alone (percent)	4.25	10.88	13.53
Resident population: two or more races (percent)	1.79	2.48	1.71
Resident population: Hispanic or Latino origin (percent)	17.58	10.44	7.41
Resident population: total females (percent)	49.70	50.41	50.93
Social security: retired workers-benefit recipients (thousands)	386.55	686.91	716.18
Corn Grain Production (dollar, millions)	492.99	1068.64	1045.22
Hay production (dollar, millions)	493.11	425.24	260.35
Farm operations (acres, millions)	62.65	85.34	34.27
Labor hired wage (per hour)	8.50	12.01	11.20
Rent cash cropland expense (acres)	60.00	64.08	75.20
Vegetable totals (dollars, millions)	110.31	158.05	140.21
Wheat production (dollars, millions)	352.67	338.87	138.08

**Table 20:** WA narrowly-defined agriculture average weekly wage per worker

	Treated	Synthetic	Sample mean
Lagged outcome	6.27	6.30	6.34
Barley for grain (acres)	245,385.00	143,746.92	41,592.84
Land in orchards (acres)	308,608.00	167,305.66	51,262.40
Snap beans harvested for sale, harvested (acres)	3418.67	12,799.15	8048.64
Fruits and nuts, cherries, tart, total acres (acres)	1976.33	3479.40	2208.25
Fruits and nuts, pears, all, total acres (acres)	26,240.67	183.71	227.10
Comm. soil conds. (thousands of treated acres)	3959.26	4957.35	5357.77
Resident population 65 years and over (percent)	11.55	12.11	13.03
Savings institutions - total deposits (thousands)	3693.15	1313.45	2692.87
Civilian labor force unemployment rate (percent)	6.50	5.21	5.53
Federal Government expenditure-grants (millions)	9.92	10.07	10.89
Federal Government insurance (millions)	7.33	54.63	25.58
Resident population: Black alone (percent)	4.45	18.64	13.53
Resident population: two or more races (percent)	2.78	1.27	1.71
Resident population: Hispanic or Latino origin (percent)	9.38	7.05	7.41
Resident population: total females (percent)	50.23	50.67	50.93
Social security: retired workers-benefit recipients (thousands)	622.15	748.42	716.18
Corn Grain Production (dollar, millions)	78.87	305.53	1045.22
Hay production (dollar, millions)	445.95	215.55	260.35
Farm operations (acres, millions)	30.02	46.90	34.27
Labor hired wage (per hour)	9.50	12.33	11.20
Rent cash cropland expense (acres)	136.50	62.55	75.20
Vegetable totals (dollars, millions)	182.97	350.80	140.21
Wheat production (dollars, millions)	782.89	255.36	138.08

**Table 21:** WA narrowly-defined agriculture total quarterly wages

	Treated	Synthetic	Sample mean
Lagged outcome	17.36	17.35	16.39
Barley for grain (acres)	245,385.00	8869.55	41,592.84
Land in orchards (acres)	308,608.00	207,300.29	51,262.40
Snap beans harvested for sale, harvested (acres)	3418.67	8706.87	8048.64
Fruits and nuts, cherries, tart, total acres (acres)	1976.33	3410.88	2208.25
Fruits and nuts, pears, all, total acres (acres)	26,240.67	378.24	227.10
Comm. soil conds. (thousands of treated acres)	3959.26	10,768.68	5357.77
Resident population 65 years and over (percent)	11.55	12.04	13.03
Savings institutions - total deposits (thousands)	3693.15	4829.62	2692.87
Civilian labor force unemployment rate (percent)	6.50	5.40	5.53
Federal Government expenditure-grants (millions)	9.92	21.16	10.89
Federal Government insurance (millions)	7.33	103.23	25.58
Resident population: Black alone (percent)	4.45	9.97	13.53
Resident population: two or more races (percent)	2.78	1.19	1.71
Resident population: Hispanic or Latino origin (percent)	9.38	22.69	7.41
Resident population: total females (percent)	50.23	50.47	50.93
Social security: retired workers-benefit recipients (thousands)	622.15	1354.89	716.18
Corn Grain Production (dollar, millions)	78.87	564.14	1045.22
Hay production (dollar, millions)	445.95	581.62	260.35
Farm operations (acres, millions)	30.02	152.02	34.27
Labor hired wage (per hour)	9.50	12.47	11.20
Rent cash cropland expense (acres)	136.50	59.11	75.20
Vegetable totals (dollars, millions)	182.97	345.46	140.21
Wheat production (dollars, millions)	782.89	161.37	138.08

**Table 22:** WA narrowly-defined agriculture average employment

	Treated	Synthetic	Sample mean
Lagged outcome	8.52	8.52	7.48
Barley for grain (acres)	245,385.00	75,321.52	41,592.84
Land in orchards (acres)	308,608.00	81,773.67	51,262.40
Snap beans harvested for sale, harvested (acres)	3418.67	8773.77	8048.64
Fruits and nuts, cherries, tart, total acres (acres)	1976.33	8330.37	2208.25
Fruits and nuts, pears, all, total acres (acres)	26,240.67	403.45	227.10
Comm. soil conds. (thousands of treated acres)	3959.26	13,251.21	5357.77
Resident population 65 years and over (percent)	11.55	11.94	13.03
Savings institutions - total deposits (thousands)	3693.15	2691.01	2692.87
Civilian labor force unemployment rate (percent)	6.50	5.78	5.53
Federal Government expenditure-grants (millions)	9.92	15.09	10.89
Federal Government insurance (millions)	7.33	30.89	25.58
Resident population: Black alone (percent)	4.45	8.54	13.53
Resident population: two or more races (percent)	2.78	1.37	1.71
Resident population: Hispanic or Latino origin (percent)	9.38	10.79	7.41
Resident population: total females (percent)	50.23	50.45	50.93
Social security: retired workers-benefit recipients (thousands)	622.15	964.12	716.18
Corn Grain Production (dollar, millions)	78.87	2217.22	1045.22
Hay production (dollar, millions)	445.95	554.67	260.35
Farm operations (acres, millions)	30.02	98.75	34.27
Labor hired wage (per hour)	9.50	8.89	11.20
Rent cash cropland expense (acres)	136.50	79.54	75.20
Vegetable totals (dollars, millions)	182.97	135.63	140.21
Wheat production (dollars, millions)	782.89	339.72	138.08

**Table 23:** WA narrowly-defined agriculture number of establishments

	Treated	Synthetic	Sample mean
Lagged outcome	5.91	5.94	4.82
Barley for grain (acres)	245,385.00	12,036.46	41,592.84
Land in orchards (acres)	308,608.00	110,787.49	51,262.40
Snap beans harvested for sale, harvested (acres)	3418.67	17,676.70	8048.64
Fruits and nuts, cherries, tart, total acres (acres)	1976.33	35,032.51	2208.25
Fruits and nuts, pears, all, total acres (acres)	26,240.67	1001.38	227.10
Comm. soil conds. (thousands of treated acres)	3959.26	5728.86	5357.77
Resident population 65 years and over (percent)	11.55	12.73	13.03
Savings institutions - total deposits (thousands)	3693.15	3733.34	2692.87
Civilian labor force unemployment rate (percent)	6.50	7.50	5.53
Federal Government expenditure-grants (millions)	9.92	14.36	10.89
Federal Government insurance (millions)	7.33	4.51	25.58
Resident population: Black alone (percent)	4.45	13.49	13.53
Resident population: two or more races (percent)	2.78	1.43	1.71
Resident population: Hispanic or Latino origin (percent)	9.38	3.78	7.41
Resident population: total females (percent)	50.23	50.86	50.93
Social security: retired workers-benefit recipients (thousands)	622.15	1062.24	716.18
Corn Grain Production (dollar, millions)	78.87	960.76	1045.22
Hay production (dollar, millions)	445.95	305.97	260.35
Farm operations (acres, millions)	30.02	22.56	34.27
Labor hired wage (per hour)	9.50	9.01	11.20
Rent cash cropland expense (acres)	136.50	74.39	75.20
Vegetable totals (dollars, millions)	182.97	161.09	140.21
Wheat production (dollars, millions)	782.89	219.83	138.08

**Table 24:** CO narrow retail average weekly wage per worker

	Treated	Synthetic	Sample mean
Lagged outcome	6.65	6.57	6.30
College graduation rate (percent)	52.48	52.41	53.63
High school graduation rate (percent)	76.25	76.77	75.35
Population density (people per square mile)	45.97	127.83	204.23
State Unemployment Rate (percent)	5.67	5.72	5.93
GDP per capita (dollars, thousands)	68.59	55.18	59.65
Tobacco Store log average weekly wage per worker	6.12	6.11	6.09

**Table 25:** CO narrow retail total quarterly wages

	Treated	Synthetic	Sample mean
Log total quarterly wages 998 lag	17.11	17.05	16.16
College graduation rate (percent)	52.48	52.45	53.63
High school graduation rate (percent)	76.25	76.28	75.35
Population density (people per square mile)	45.97	99.35	204.23
State unemployment rate (percent)	5.67	5.66	5.93
GDP per capita (dollars, thousands)	68.59	62.11	59.65
Tobacco Store log total quarterly wages	14.78	14.76	14.66

**Table 26:** CO narrow retail average employment

	Treated	Synthetic	Sample mean
Log average employment 998 lag	7.90	7.85	7.31
College graduation rate (percent)	52.48	52.44	53.63
High school graduation rate (percent)	76.25	76.28	75.35
Population density (people per square mile)	45.97	87.94	204.23
State unemployment rate (percent)	5.67	5.48	5.93
GDP per capita (dollars, thousands)	68.59	63.53	59.65
Tobacco Store log average employment	6.10	6.10	6.02

**Table 27:** CO narrow retail number of establishments

	Treated	Synthetic	Sample mean
Log number of establishments 998 lag	6.28	6.27	5.73
College graduation rate (percent)	52.48	52.54	53.63
High school graduation rate (percent)	76.25	76.04	75.35
Population density (people per square mile)	45.97	84.01	204.23
State unemployment rate (percent)	5.67	5.49	5.93
GDP per capita (dollars, thousands)	68.59	61.31	59.65
Tobacco Store log number of establishments	4.77	4.74	4.47

**Table 28:** WA narrow retail average weekly wage per worker

	Treated	Synthetic	Sample mean
Lagged outcome	6.32	6.33	6.30
College graduation rate (percent)	63.07	59.27	53.63
High school graduation rate (percent)	73.58	73.90	75.35
Population density (people per square mile)	95.95	158.82	204.23
State unemployment rate (percent)	6.90	6.43	5.93
GDP per capita (dollars, thousands)	70.29	57.73	59.65
Tobacco Store log average weekly wage per worker	6.03	6.20	6.09

**Table 29:** WA narrow retail total quarterly wages

	Treated	Synthetic	Sample mean
Log total quarterly wages 998 lag	16.09	16.07	16.16
College graduation rate (percent)	63.07	59.18	53.63
High school graduation rate (percent)	73.58	73.89	75.35
Population density (people per square mile)	95.95	362.27	204.23
State unemployment rate (percent)	6.90	6.69	5.93
GDP per capita (dollars, thousands)	70.29	69.04	59.65
Tobacco Store log total quarterly wages	14.52	14.71	14.66

**Table 30:** WA narrow retail average employment

	Treated	Synthetic	Sample mean
Log average employment 998 lag	7.20	7.24	7.31
College graduation rate (percent)	63.07	59.31	53.63
High school graduation rate (percent)	73.58	75.02	75.35
Population density (people per square mile)	95.95	342.54	204.23
State unemployment rate (percent)	6.90	6.68	5.93
GDP per capita (dollars, thousands)	70.29	68.95	59.65
Tobacco Store log average employment	5.93	6.11	6.02

**Table 31:** WA narrow retail number of establishments

	Treated	Synthetic	Sample mean
log number of establishments 998 lag	5.63	6.14	5.73
College graduation rate (percent)	63.07	59.52	53.63
High school graduation rate (percent)	73.58	78.49	75.35
Population density (people per square mile)	95.95	144.57	204.23
State unemployment rate (percent)	6.90	6.26	5.93
GDP per capita (dollars, thousands)	70.29	57.48	59.65
Tobacco Store log number of establishments	4.97	4.94	4.47

**Table 32:** CO broad retail average weekly wage per worker

	Treated	Synthetic	Sample mean
Lagged outcome	6.25	6.25	6.26
College graduation rate (percent)	52.48	52.72	53.63
High school graduation rate (percent)	76.25	76.39	75.35
Population density (people per square mile)	45.97	126.41	204.23
State unemployment rate (percent)	5.67	5.76	5.93
GDP per capita (dollars, thousands)	68.59	59.97	59.65

**Table 33:** CO broad retail total quarterly wages

	Treated	Synthetic	Sample mean
Lagged outcome	20.04	20.04	20.14
College graduation rate (percent)	52.48	48.54	53.63
High school graduation rate (percent)	76.25	76.28	75.35
Population density (people per square mile)	45.97	68.04	204.23
State unemployment rate (percent)	5.67	5.73	5.93
GDP per capita (dollars, thousands)	68.59	56.62	59.65

**Table 34:** CO broad retail average employment

	Treated	Synthetic	Sample mean
Lagged outcome	11.23	11.23	11.31
College graduation rate (percent)	52.48	47.95	53.63
High school graduation rate (percent)	76.25	76.70	75.35
Population density (people per square mile)	45.97	55.57	204.23
State unemployment rate (percent)	5.67	5.67	5.93
GDP per capita (dollars, thousands)	68.59	56.81	59.65

**Table 35:** CO broad retail number of establishments

	Treated	Synthetic	Sample mean
Lagged outcome	8.33	8.33	8.43
College graduation rate (percent)	52.48	56.25	53.63
High school graduation rate (percent)	76.25	76.25	75.35
Population density (people per square mile)	45.97	381.19	204.23
State unemployment rate (percent)	5.67	5.79	5.93
GDP per capita (dollars, thousands)	68.59	68.52	59.65

**Table 36:** WA broad retail average weekly wage per worker

	Treated	Synthetic	Sample mean
Lagged outcome	6.40	6.40	6.26
College graduation rate (percent)	63.07	53.43	53.63
High school graduation rate (percent)	73.58	70.26	75.35
Population density (people per square mile)	95.95	264.90	204.23
State unemployment rate (percent)	6.90	5.98	5.93
GDP per capita (dollars, thousands)	70.29	70.85	59.65

**Table 37:** WA broad retail total quarterly wages

	Treated	Synthetic	Sample mean
Lagged outcome	20.43	20.43	20.14
College graduation rate (percent)	63.07	60.78	53.63
High school graduation rate (percent)	73.58	73.94	75.35
Population density (people per square mile)	95.95	180.77	204.23
State unemployment rate (percent)	6.90	5.17	5.93
GDP per capita (dollars, thousands)	70.29	63.21	59.65

**Table 38:** WA broad retail average employment

	Treated	Synthetic	Sample mean
Lagged outcome	11.47	11.47	11.31
College graduation rate (percent)	63.07	59.88	53.63
High school graduation rate (percent)	73.58	73.84	75.35
Population density (people per square mile)	95.95	146.39	204.23
State unemployment rate (percent)	6.90	5.87	5.93
GDP per capita (dollars, thousands)	70.29	58.93	59.65

**Table 39:** WA broad retail number of establishments

	Treated	Synthetic	Sample mean
Lagged outcome	8.48	8.48	8.43
College graduation rate (percent)	63.07	59.59	53.63
High school graduation rate (percent)	73.58	73.87	75.35
Population density (people per square mile)	95.95	396.66	204.23
State unemployment rate (percent)	6.90	6.50	5.93
GDP per capita (dollars, thousands)	70.29	70.13	59.65

## Data Availability

All data are taken from publicly available United States government sources.

## Abbreviations

BLS: Bureau of Labor Statistics
CART: classification and regression trees
CBD: cannabidiol
GDP: gross domestic product
LASSO: least absolute shrinkage and selection operator
NAICS: North American Industry Classification System
RMSPE: ratio of the mean squared prediction errors
THC: tetrahydrocannbinol
US: United States
